# An Extended Hydro-Mechanical Coupling Model Based on Smoothed Particle Hydrodynamics for Simulating Crack Propagation in Rocks under Hydraulic and Compressive Loads

**DOI:** 10.3390/ma16041572

**Published:** 2023-02-13

**Authors:** Dianrui Mu, Aiping Tang, Haigang Qu, Junjie Wang

**Affiliations:** 1 School of Civil Engineering, Harbin Institute of Technology, Harbin 150090, China; 2 Department of Civil Engineering, University of Ottawa, Ottawa, ON K1N6N5, Canada

**Keywords:** smoothed particle hydrodynamics (SPH), seepage model, hydro-mechanical coupling, osmotic pressure, crack propagation

## Abstract

A seepage model based on smoothed particle hydrodynamics (SPH) was developed for the seepage simulation of pore water in porous rock mass media. Then, the effectiveness of the seepage model was proved by a two-dimensional seepage benchmark example. Under the framework of SPH based on the total Lagrangian formula, an extended hydro-mechanical coupling model (EHM-TLF-SPH) was proposed to simulate the crack propagation and coalescence process of rock samples with prefabricated flaws under hydraulic and compressive loads. In the SPH program, the Lagrangian kernel was used to approximate the equations of motion of particles. Then, the influence of flaw water pressure on crack propagation and coalescence models of rock samples with single or two parallel prefabricated flaws was studied by two numerical examples. The simulation results agreed well with the test results, verifying the validity and accuracy of the EHM-TLF-SPH model. The results showed that with the increase in flaw water pressure, the crack initiation angle and stress of the wing crack decreased gradually. The crack initiation location of the wing crack moved to the prefabricated flaw tip, while the crack initiation location of the shear crack was far away from the prefabricated flaw tip. In addition, the influence of the permeability coefficient and flaw water pressure on the osmotic pressure was also investigated, which revealed the fracturing mechanism of hydraulic cracking engineering.

## 1. Introduction

Hydraulic cracking, as a practical science and technology, has been extended to the energy exploitation of underground engineering, and some satisfactory engineering results have been achieved. However, the application of hydraulic cracking technology in complex discontinuous geological conditions still faces some engineering problems, such as the difficulty in controlling the propagation direction of cracks caused by hydraulic cracking and the environmental pollution caused by improper fracturing schemes. Therefore, in order to improve the exploitation efficiency of underground energy and avoid man-made damage to the environment, it is key to apply hydraulic cracking technology in practical engineering to accurately simulate the crack evolution of deep rock mass under flaw water pressure. In order to more accurately reveal the coupling mechanism of hydraulic cracking, researchers have implemented a large amount of numerical work and simulated the crack propagation process in fractured rock mass under the condition of hydraulic–mechanical coupling.

The finite element method (FEM), as an efficient mesh numerical method, is often used to investigate the crack propagation and coalescence behavior of rocks. Liu et al. [1] established an FEM model embedded with zero-thickness cohesion elements to simulate and analyze the spatial distribution of cracks driven by hydraulic cracking. Silva and Einstein [2] studied the initiation of cracks in rock samples with prefabricated defects under different fracture water pressures and vertical stresses based on FEM. Robert [3] simulated the dynamic process of the hydraulic cracking of rocks by coupling peridynamic theory with FEM. However, FEM has the limitation that the cracks should overlap with the meshed elements, which makes the mesh generation very difficult. Especially, singular mesh elements frequently occur at the tip of the prefabricated flaw after the mesh elements are divided, making it impossible to simulate arbitrary crack propagation [4].

The numerical manifold method (NMM) was developed from FEM and discontinuous deformation analysis (DDA) [5]. In recent years, NMM has been extended to the hydraulic cracking of rocks by many researchers. Xu et al. [6] developed a grouting reinforcement simulator based on NMM to simulate the migration and solidification process of grout in a rock fracture network. In order to investigate the interaction between unsaturated soil and water at microscale, Sun et al. [7] proposed an extended NMM by introducing a soil–water coupling model that considered the capillary force calculation and capillary water distribution. The cohesive element-based NMM with Voronoi grains was extended by Wu et al. [8] to study the hydraulic fracture process of rock by embedding a coupled hydro-mechanical model. However, when the flaw tip is located inside the grid element, the numerical accuracy of NMM decreases, requiring a regular mathematical coverage system [9] to accurately evaluate the stress intensity factors (SIFs).

In order to accurately simulate the crack propagation process in rocks under hydro-mechanical coupling, meshless methods such as the discrete element method (DEM), peridynamic (PD) theory, and smoothed particle hydrodynamics (SPH) have been extended to geotechnical engineering. Based on computational fluid dynamics, Ajayi et al. [10] established a discrete model to study the effects of different radon control measures on cave mines. A hydraulic–mechanical coupling grouting simulator was developed by Liu and Sun [11] for simulating the fracture grouting process by combining the finite element method (FEM) and DEM. Based on FEM and DEM, a hydro-mechanical coupling model (Y-grouting) was proposed by Sun et al. [12] to investigate the grouting process. Based on the effective stress theory, Helmons et al. [13] established a coupling model of DEM and pore pressure diffusion, successfully predicting the strengthening/weakening of the rock. However, there are still some problems when DEM is used to solve continuous problems [14]. In order to establish the relationship between macroparameters and microparameters, DEM requires tedious parameter calibration [15]. In addition, the cracks obtained by DEM can only propagate along the contact surfaces between discrete elements, and the motion equations used by DEM are in differential forms without spatial derivatives, resulting in discontinuous displacement of the crack tips.

Peridynamic theory was originally proposed by Silling [16] based on the idea of non-local influence domains, including ordinary state-based peridynamics, non-ordinary state-based peridynamics, and bond-based peridynamics. Zhang et al. [17] proposed an extended ordinary state-based peridynamic model to simulate the fluid–structure coupling and hydraulic fracturing and successfully simulated the crack propagation process and the fluid flow behavior along the crack. Zhou et al. [18] proposed a fully coupled hydro-mechanical model based on bond-based peridynamics to simulate the hydraulic fracturing process of fractured rock masses. Shou [19] proposed a coupled hydro-mechanical state-based peridynamics for simulating the failure process of complete rock samples under biaxial compression. Although peridynamics has been successfully used to simulate hydraulic cracking of rocks and achieved satisfactory numerical results, the assumption of pairwise forces in bond-based peridynamics limits the Poisson’s ratio to be 1/4 for isotropic problems. To address this problem, Zhou and Shou [20] introduced tangential bonds in bond-based peridynamics and achieved good results. However, the method reduced the computational efficiency, making it difficult to be used for 3D numerical computation with hundreds of thousands of particles.

In order to solve astrophysics problems, the SPH method was first proposed by Ginold and Monaghan [21] and Lucy [22]. With the rapid development of SPH, it is widely used in more practical engineering problems, such as fluid mechanics [23,24], heat conduction [25,26,27], and blasting-induced rock cracking [28,29,30]. In addition, SPH has been widely used in numerical studies related to hydro-mechanical coupling. Based on the general particle dynamics (GPD), Bi and Zhou [4] considered the interaction between solid and liquid particles and successfully simulated the crack propagation and coalescence of rock samples with the prefabricated flaws under the flaw water pressure and compressive loads. To further reveal the interaction mechanism between hydraulic fractures (HFs) and natural fractures (NFs), Yu et al. [31] proposed a 2P-IKSPH meshless numerical method based on the improved kernel SPH method. Although the above method can accurately describe the solid–liquid contact force on the crack in any direction, the procedure is time-consuming. In order to solve this problem, Mu et al. [32] proposed a new fluid–structure interaction model under the Lagrangian framework, which did not need to calculate the solid–liquid contact force, significantly improving the computational efficiency of the program. However, this model did not consider the effect of the osmotic pressure of fractured water on the initiation and propagation of rock mass with pre-existing flaws.

In this paper, an accurate and effective meshless numerical method considering hydro-mechanical damage coupling was proposed to simulate the crack propagation and coalescence process of rocks with the prefabricated flaws under compression and flaw water pressure. First, according to Darcy’s law and phase field theory, the calculation formula of the permeability coefficient of porous rock mass media under the coupling condition of hydro-mechanical damage was proposed, and the seepage model of porous rock mass media under this condition was established based on the SPH method. Second, based on Biot fluid–structure interaction theory, a hydro-mechanical damage coupling model of porous rock mass media considering the effect of osmotic pressure was established under the framework of TLSPH. The advantage of the EHM-TLF-SPH model was that the implementation strategy of hydro-mechanical damage coupling did not involve the interface processing, which not only simplified the programming significantly but also avoided the non-physical penetration of two-phase particles at the interface. In addition, the present model adopted the idea of a “virtual bond” proposed by Zhou Xiaoping’s group [4,27,33] under the framework of generalized particle dynamics (GPD), that is, the interactions between neighboring particles are transmitted through virtual links. Once the stress on the virtual link reaches its ultimate strength, the virtual link breaks, and the interaction between the particles disappears. Finally, the accuracy of the EHM-TLF-SPH model was verified by numerical examples, the results of which agreed well with the experimental results.

## 2. Seepage Model of Fractured Rock Mass Based on SPH

The seepage model of fractured rock mass includes an equivalent continuous media seepage model, a discontinuous media seepage model of a fractured network, and a two-phase media seepage model. The equivalent continuum seepage model has been widely used in seepage simulation, and satisfactory numerical results have been obtained. The model is based on the permeability tensor, and the fractured rock mass is simplified as the continuum to solve the seepage problem [34]. Therefore, based on the equivalent continuum seepage model, the relationship between permeability coefficient and damage of fractured rock mass will be established in this section under the SPH framework to describe the seepage characteristics of pore water in fractured rock mass.

### 2.1. Seepage Control Equation of Fractured Rock Mass and Its Discretization

**Figure 1:**
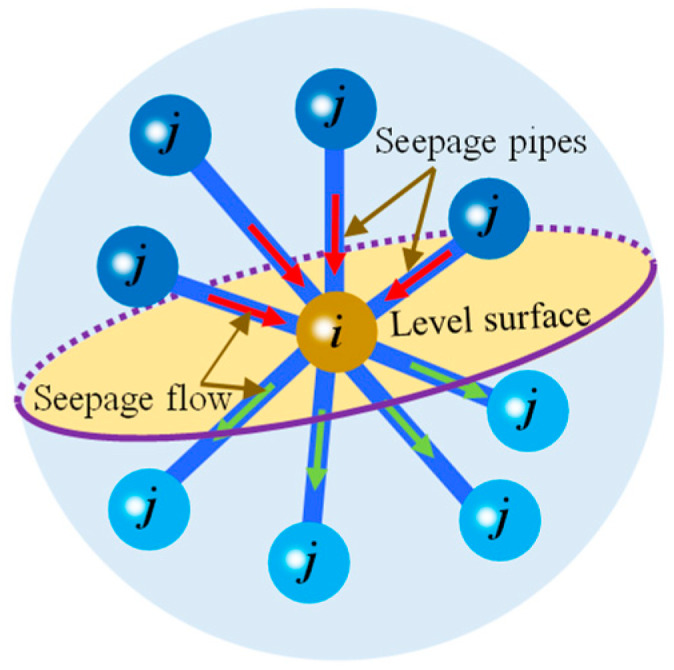
The interacting particles transfer water flow through virtual links.

Assume that particle *i* is a water flow reservoir, and the virtual link has dual characteristics in hydraulic conduction. It can not only establish the interaction between particles but also serve as a seepage pipe to transfer water flow between interacting particles. In the seepage system shown in Figure 1, the water flow reservoir and seepage pipe are necessary conditions for the hydraulic conduction of pore water in porous rock mass media.

According to the seepage theory, the water flow in porous rock mass media conforms to Darcy’s law, then the seepage control equation under isotropic conditions is

(1) $$S\cdot \frac{\partial H}{\partial t}=\nabla \cdot \left(\kappa \;\nabla H\right)$$

where *H* is the head; κ is the permeability coefficient; $S=\rho_{w}𝘨(\alpha +n\beta )$ is specific yield; $\rho_{w}$ is the density of pore water; *α* and *n* are the compressibility and porosity of porous rock mass media, respectively; and *β* is the compressibility of pore water.

Based on Darcy’s law, the velocity of pore water in porous rock mass media can be obtained as follows:

(2) $$v_{w}=-\kappa \nabla H$$

In order to further improve the computation accuracy of head gradient in Equation (1), the seepage control equation needs to be calculated using a step-by-step discrete format. Substituting Equation (2) into Equation (1), the water head change rate can be expressed as

(3) $$\frac{\partial H}{\partial t}=-\frac{1}{S}\nabla \cdot v_{w}$$

According to the particle approximation method of SPH, Equations (2) and (3) can be discretized as follows:

(4) $$\left\{\begin{array}{l} v_{w,i}=\kappa_{i}\sum_{j}\frac{m_{j}}{\rho_{j}}\left(H_{i}-H_{j}\right)\frac{\partial W_{ij}}{\partial x_{i}}\\ \frac{\partial H_{i}}{\partial t}=\frac{1}{S_{i}}\sum_{j}\frac{m_{j}}{\rho_{j}}\left(v_{w,i}-v_{w,j}\right)\frac{\partial W_{ij}}{\partial x_{i}} \end{array}\right.$$

where $\partial W_{ij}/\partial x_{i}$ is the kernel function gradient; $H_{i}$ and $H_{j}$ denote the head of particles *i* and *j*, respectively; and $v_{w,i}$ and $v_{w,j}$ are the seepage velocity of particles *i* and *j*, respectively.

Shou [19] established a 2D permeability tensor model for fractured rock mass and derived the expression of equivalent continuous permeability coefficient of porous rock mass medium and permeability coefficient of macroscopic flaw surface as follows:

(5) $$\left\{\begin{array}{l} \kappa_{s}=\frac{\gamma_{w}\lambda b_{0}^{3}}{24\eta }=\frac{𝘨\lambda b_{0}^{3}}{24u}\\ \kappa_{d}=\frac{\gamma_{w}b_{0}^{2}}{12\eta }=\frac{𝘨b_{0}^{2}}{12u} \end{array}\right.$$

where *λ* is the number of prefabricated defects per unit length, *b*_0_ is the initial opening of the prefabricated defect surface, and *u* is the kinematic viscosity of water.

**Figure 2:**
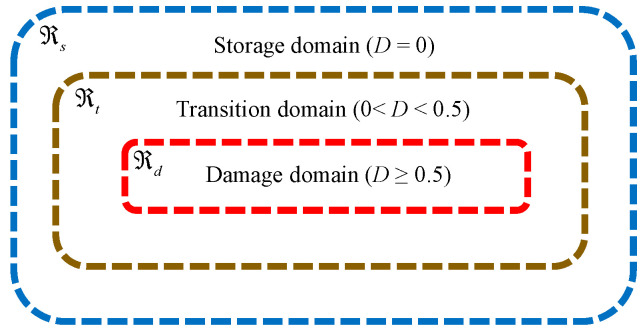
Seepage domain of porous rock mass media [35,36].

In order to simulate the seepage process of fractured rock mass, porous rock mass media can be divided into the storage domain, transition domain, and damage domain according to the damage degree of the material particles. The transition domain is a computing domain between the storage domain and damage domain, as shown in Figure 2.

**Figure 3:**
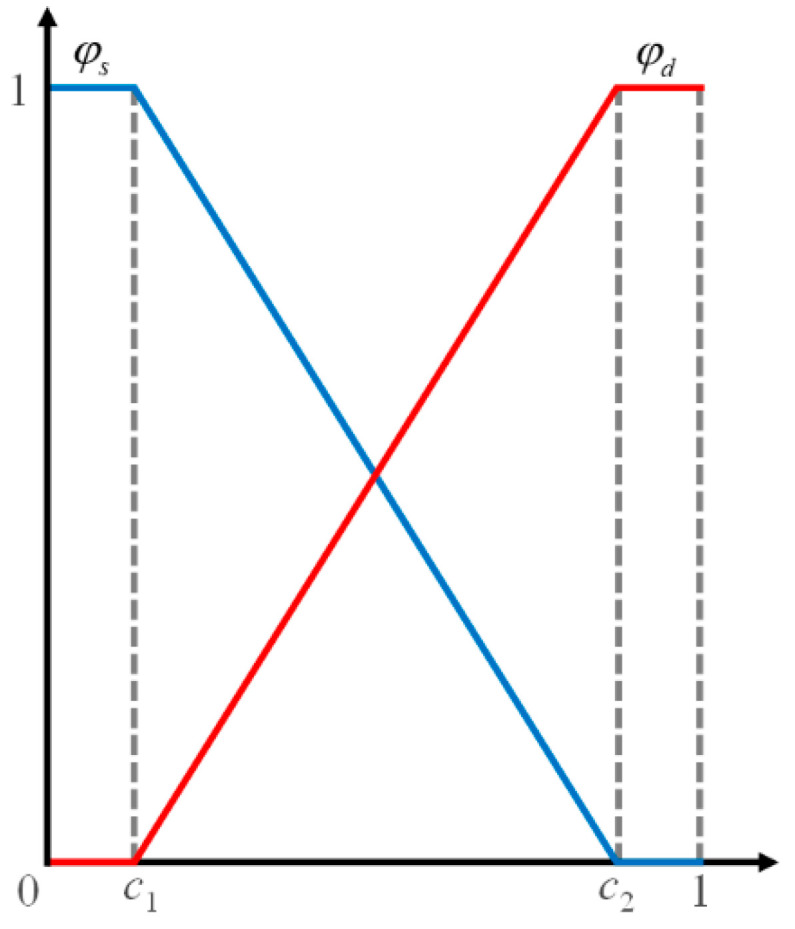
Linear interpolation function of the seepage model [35,36].

As shown in Figure 3, according to the phase field theory [35], the linear interpolation functions $\psi_{d}$ and $\psi_{s}$ are introduced to establish the relationship between the storage domain, transition domain, and damage domain as follows:

(6) $$\left\{\begin{array}{l} \psi_{d}=\frac{D-c_{1}}{c_{2}-c_{1}}\\ \psi_{s}=1-\psi_{d} \end{array}\right.$$

where *D* is the damage function and *c*_1_ and *c*_2_ are the upper and lower thresholds of the damage function, respectively.

In smoothed particle hydrodynamics, only the influence of the permeability coefficient on pore water seepage is considered. Assuming that the virtual links between interacting particles can be regarded as seepage pipes, after introducing linear interpolation functions $\psi_{d}$ and $\psi_{s}$, the permeability coefficient of virtual links in porous rock mass medium with prefabricated flaws can be written in the general form as follows:

(7) $$\kappa_{t}=\psi_{d}\kappa_{d}+\psi_{s}\kappa_{s}$$

where $\kappa_{d}$ and $\kappa_{s}$ are the permeability coefficients of the damage domain and storage domain, respectively. When $\psi_{s}$ = 0 and $\psi_{d}$ = 1, $\kappa_{t}$ represents the permeability coefficient of the damage domain. When 0 < $\psi_{s}$ < 1 and 0 < $\psi_{d}$ < 1, $\kappa_{t}$ represents the permeability coefficient of the transition domain. When $\psi_{s}$ = 1 and $\psi_{d}$ = 0, $\kappa_{t}$ represents the permeability coefficient of the storage domain.

In the seepage model, water flow is transmitted through virtual links, and the permeability coefficient of the virtual link can be obtained by averaging the permeability coefficients of two interacting particles:

(8) $$\kappa_{t,ij}=(\kappa_{t,i}+\kappa_{t,j})/2$$

According to Equations (4)–(8), the discrete formula of the seepage control equation under the coupled condition of hydro-mechanical damage can be obtained as follows:

(9) $$\left\{\begin{array}{l} \psi_{d,i}=\frac{D_{i}-c_{1}}{c_{2}-c_{1}},\;\psi_{s,i}=1-\psi_{d,i}\;\\ \kappa_{t,i}=\;\frac{𝘨b_{0}^{2}(\lambda b_{0}\psi_{s,i}+2\psi_{d,i})}{24u}\\ v_{w,i}=\frac{\kappa_{t,i}+\kappa_{t,j}}{2}\sum_{j}\frac{m_{j}}{\rho_{j}}(H_{i}-H_{j})\frac{\partial W_{ij}}{\partial x_{i}}\\ \frac{\partial H_{i}}{\partial t}=\frac{1}{S_{i}}\sum_{j}\frac{m_{j}}{\rho_{j}}(v_{w,i}-v_{w,j})\frac{\partial W_{ij}}{\partial x_{i}} \end{array}\right.$$

### 2.2. Benchmark Example—Two-Dimensional Seepage Simulation of Intact Rock Sample

**Figure 4:**
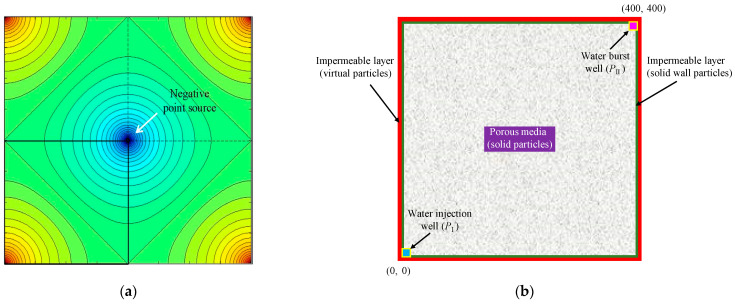
Two-dimensional seepage model for an intact rock sample. (**a**) Schematic diagram of the five-point well network, reprinted from Ref. [38]. (**b**) Geometric model.

In order to demonstrate the validity of the seepage model, a square sample of 400 m × 400 m in size was selected for the 2D seepage numerical simulation [37]. Figure 4a shows a schematic diagram of a five-spot well network [38]. The high-pressure water injected from the four water injection wells at the corners of the five-spot well network provides a strong driving force for the seepage of oil in the rock mass, so that the oil to be recovered is gushed out from the middle well. Figure 4b shows a simplified geometric model of the five-point well network. The initial water pressure was 0, and the water pressures of the water injection well and water burst well were *P*_I_ = 8 MPa and *P*_II_ = −8 MPa, respectively.

In addition, the four boundaries of the rock sample were composed of solid wall particles (I-type virtual particles), and the impermeable layers of the four boundaries of the model were represented by ghost virtual particles (II-type virtual particles). The pore water pressure of the ghost particles and that of the solid particles were symmetrical about the solid boundary. The numerical model was represented by 200 × 200 = 40,000 particles, and the distance between adjacent particles was Δ*x* = 2.0 m. The seepage integration step was Δt*^h^* = 1.0 × 10^−3^ d, and the influence domain radius was *δ* = 3.015d*x*. The 2D seepage simulation parameters of a square intact rock sample are shown in Table 1.

**Figure 5:**
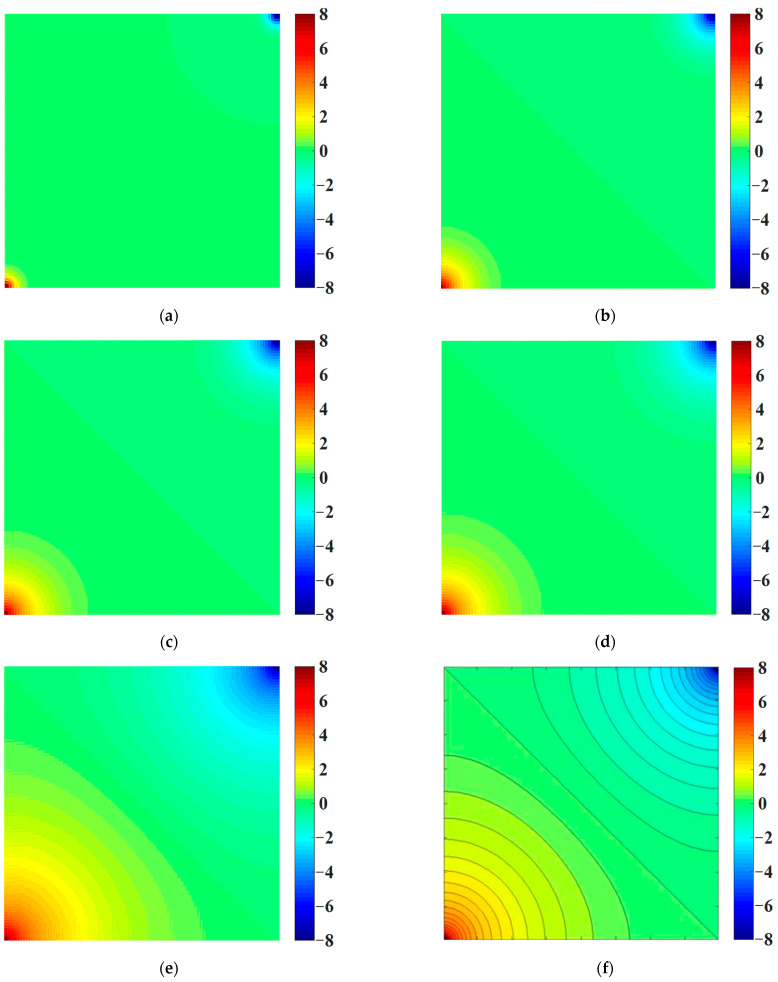
Cloud map of water pressure distribution for the intact sample at different seepage times (unit: MPa). (**a**) t = 0.1 d. (**b**) t = 1 d. (**c**) t = 2 d. (**d**) t = 3 d. (**e**) t = 100 d. (**f**) Analytical solution [38].

Figure 5 shows the cloud map of water pressure distribution for the intact sample at different seepage times. It can be seen from Figure 5 that the water pressure gradient and seepage velocity at the water injection well and water burst well were larger and faster than those at other locations in the model. When the seepage time was t = 1 d, an obvious water pressure demarcation line appeared in the model, and the magnitude of the water pressure for the water injection well and water burst well was symmetrically distributed with respect to the demarcation line. With the increase in seepage time, the pressure gradient at the water injection well and water burst well gradually decreased, and the seepage velocity gradually decreased. As shown in Figure 5e,f, when the seepage time was t = 100 d, the seepage field tended to be stable, and the numerical results agreed well with the analytical solutions [38] of the water pressure under the two-dimensional seepage steady distribution, verifying the effectiveness of the seepage model in solving the two-dimensional seepage problem.

**Figure 6:**
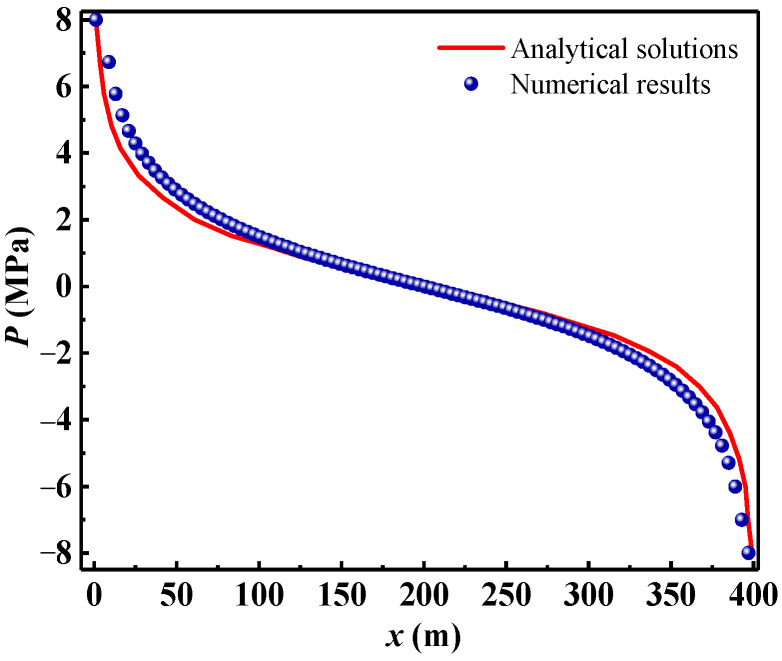
Numerical and analytical solutions for the water pressure of particles located on the diagonal line of *y* = *x*.

In order to further verify the accuracy of the seepage model, the water pressure of the particles located on the diagonal line of the seepage model was extracted for analysis and comparison. Figure 6 shows the numerical and analytical solutions for the water pressure of the particles located on the diagonal line of *y* = *x*. As can be seen from Figure 6, the numerical results agreed well with the analytical solutions [38] on the whole. Since the assumptions of the seepage model are not completely consistent with those of the analytical solution, and the water injection well and water burst well were discretized into fewer particles (6 × 6 particles), respectively, the numerical results were not completely consistent with the analytical solution. Thus, the numerical solution of the pore water pressure at 100 m near the water injection well and water burst well was slightly larger than the analytical solution.

### 2.3. Two-Dimensional Seepage Characteristics for a Rock Sample with a Prefabricated Horizontal Penetrating Flaw

**Figure 7:**
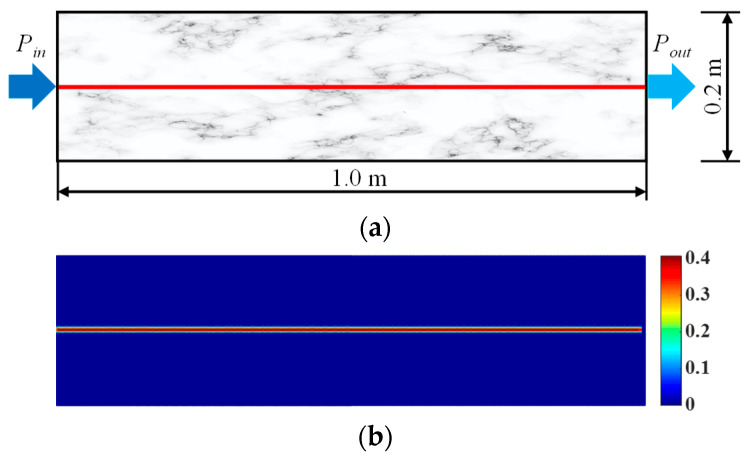
A two-dimensional seepage model for a rock sample with a prefabricated horizontal penetrating flaw. (**a**) Geometric model. (**b**) Numerical model.

In order to investigate the seepage characteristics of pore water in porous rock mass medium with prefabricated flaws, a rock sample with a prefabricated horizontal penetrating flaw was numerically simulated. The geometric dimensions and boundary conditions of the model were the same as those in reference [36,39]. As shown in Figure 7a, the size of the rock sample was length × width = 1.0 m × 0.2 m, and the pressures applied on the left and right boundaries of the model were *P_in_* = 9.5 MPa and *P_out_* = 4.5 MPa, respectively. Driven by the water pressure gradient, the pore water flowed uniformly from left to right. The prefabricated horizontal penetrating flaw with a width of 0.025 mm could be obtained by the prefabricated node segment method [40], as shown in Figure 7b. The numerical model was represented by 400 × 400 = 40,000 particles, and the distance between adjacent particles was Δ*x* = 5.0 × 10^−3^ m. The seepage integration step was Δt*^h^* = 1.0 × 10^−7^ s, and the influence domain radius was δ = 3.015d*x*. The seepage simulation parameters for a rock sample with a prefabricated horizontal penetrating flaw are given in Table 2.

**Figure 8:**
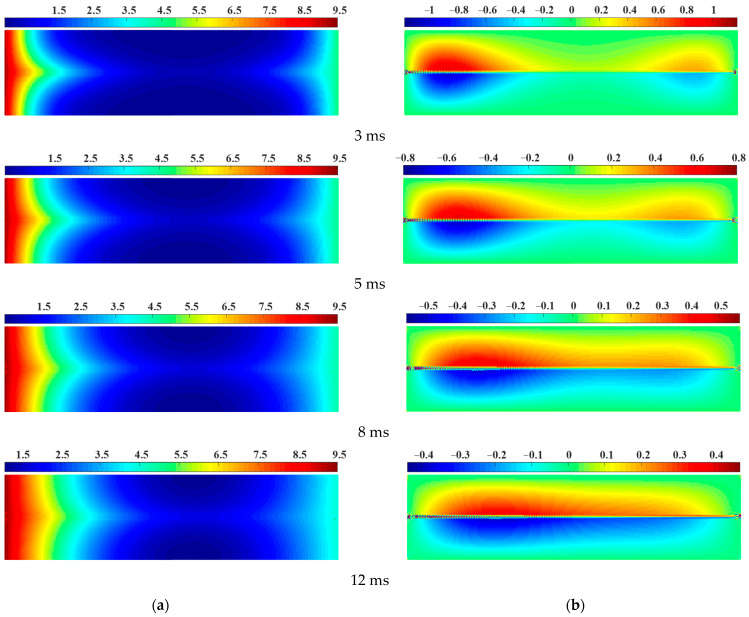
Seepage process of a sample with a prefabricated horizontal penetrating flaw at different times. (**a**) Pore water pressure. (**b**) Vertical seepage velocity.

The seepage process of a sample with a prefabricated horizontal penetrating flaw at different times was presented in Figure 8. Since the water pressure of the initial model was 0, after the water pressure boundary condition was applied to the model, convection occurred in the pore water from both ends to the middle driven by the water pressure gradient (see Figure 8a). The seepage velocity at the left end of the model was significantly greater than that at the right end because of the relatively large water pressure applied at the left end. The broken virtual links accelerated the flow of the pore water in the seepage pipe, so the pore water in the horizontal flaw had been in an advanced diffusion state during the entire seepage process. As time passed, the pore water flowing out from both sides of the prefabricated flaw met first, with the diffusion velocity of the pore water in the right flaw further suppressed, as shown in Figure 8a. In addition, it could be seen from Figure 8b that the vertical seepage velocity of pore water reached the maximum on the upper and lower sides of the prefabricated flaw and decreased uniformly from the middle to the upper and lower sides, roughly presenting an asymmetric “dumbbell-shaped” distribution. When the seepage time was t = 5 ms, the vertical seepage velocity of pore water on the left and right sides began to merge to the middle. When the seepage time was t = 12 ms, the vertical seepage velocity of pore water on the left and right sides were uniformly merged, and the asymmetric dumbbell shape completely disappeared. The numerical results showed that the two-dimensional seepage model could well reveal the seepage characteristics of pore water in porous rock mass medium with prefabricated flaws.

## 3. Extended Hydro-Mechanical Coupling Model Based on TLF-SPH

The stress field and damage state of rock mass affect the permeability coefficient of rock mass by changing the porosity of the rock mass skeleton. The change of the permeability coefficient of the rock mass will cause the change of the pore water pressure and then affect the stress field of the rock mass. In this section, the influence of the stress field on the seepage field will be realized by obtaining the calculation formula of the permeability coefficient considering the effective stress and damage effect. Meanwhile, according to Biot’s fluid–structure coupling theory, the pore water pressure and osmotic pressure terms are introduced into the constitutive equation and momentum equation, respectively, to describe the influence of seepage field on stress field.

### 3.1. Hydro-Mechanical Coupling Constitutive Equation

**Figure 9:**
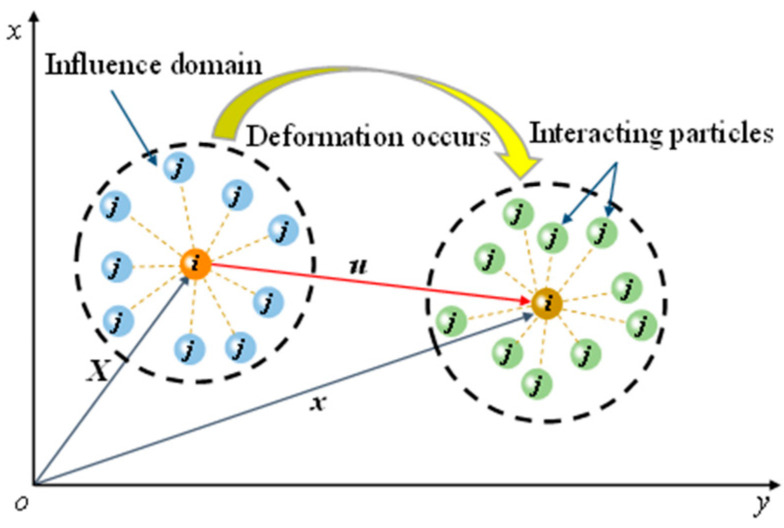
Deformation diagram of particle *i* under external force load, adapted from Ref. [25].

Based on Fourier analysis, Belytschko et al. [41] analyzed the stability of SPH methods by using different solving schemes. The results showed that the stability of the SPH method with Euler and Lagrangian kernels was obviously different, and the Lagrangian kernel can avoid the tensile instability in the SPH method, thereby improving the numerical accuracy. Therefore, under the Lagrangian framework, a hydro-mechanical coupling constitutive equation in total Lagrangian form will be developed in this section. Figure 9 shows the deformation diagram of particle *i* under the external force load. In the initial configuration *R*_0_, there was a particle *i* with a position vector ***X***. Driven by the external load, particle *i* deformed in the configuration *R_t_* with a position vector ***x***. Then, the relative position vector of particle *i* after deformation was ***u*** = ***x***−***X***.

Based on the theory of continuum mechanics, the Lagrangian form of the displacement gradient and deformation gradient tensors can be expressed as [42]

(10) $$\left\{\begin{array}{l} L=\frac{du}{dX}\\ F=\frac{dx}{dX} \end{array}\right.$$

where ***X*** is the position vector of the particle in the initial configuration; ***x*** is the position vector of the particle in the deformation configuration. ***u*** is the relative position vector of the particle before and after deformation, and ***u*** = ***x***−***X***.

Then, the Green–Lagrange strain tensor can be obtained from the displacement gradient tensor:

(11) $$E=\frac{1}{2}(L^{T}+L+L^{T}L)$$

Based on the deformation gradient and Green–Lagrange strain tensors, the Euler strain tensor can be obtained:

(12) $$\varepsilon =F^{-T}EF^{T}$$

where the superscript “−T” denotes the transpose of the inversion of matrix F, and the superscript “T” denotes the transpose of matrix F.

According to Biot fluid–structure interaction theory [43], the hydro-mechanical coupling constitutive relationship of porous rock mass medium is

(13) $$\sigma (x,p,t)=2G\varepsilon (x,t)+\left\{\lambda tr\left[\varepsilon (x,t)\right]+\alpha p\right\}I$$

where *tr*[] is the trace operator for matrices, *α* is the effective-stress coefficient, *p* is the hydrostatic pressure, *G* is the shear modulus, and *λ* is the Lame constant.

Then, the first kind of Piola–Kirchhoff stress tensor can be obtained by

(14) $$P(x,p,t)=\det\left[F(x,t)\right]\sigma (x,p,t)F^{-T}(x,t)$$

### 3.2. Hydro-Mechanical Coupling Momentum Equation Considering the Osmotic Pressure Effect

**Figure 10:**
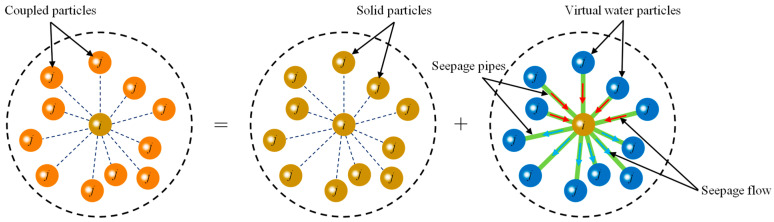
Dual characterization of coupled particles *i* under osmotic pressure.

When pore water flows in porous rock mass media, the viscous flow of pore water causes a driving force called osmotic pressure that moves solid particles. According to Section 2, the seepage model can accurately simulate the flow of pore water in porous rock mass media. However, the solid particles in the model do not actually move during the entire seepage process of pore water. In other words, the flow of pore water in the seepage model is a virtual numerical representation. Therefore, each model particle has the characteristics of water particles such as water pressure, flow velocity, etc., which provides the possibility for the calculation of the osmotic pressure of solid particles. In order to more clearly describe the dual characteristics of model particles, solid particles with fluid properties in the seepage field are defined as virtual water particles, while model particles with both solid and fluid properties are called coupled particles, as shown in Figure 10. In addition, the virtual link between the coupled particles can serve not only as the seepage pipe between interacting particles but also as a medium for osmotic pressure transfer between solid particle *i* and virtual water particle *j*.

After osmotic pressure is introduced into the momentum equation, the momentum equation of solid particles (without considering the body force) can be expressed as follows:

(15) $$\frac{dv(x,p,t)}{dt}=\frac{1}{\rho_{0}}\frac{\partial P(x,p,t)}{\partial x}+\frac{F_{s}}{\rho_{0}}$$

where $F_{s}$ is the osmotic pressure.

According to Darcy’s law and Biot’s mixture theory, the osmotic pressure acting on solid particles and the osmotic reaction force acting on virtual water particles can be obtained as follows [44,45]:

(16) $$\left\{\begin{array}{l} F_{s}=n^{2}\frac{\rho_{w}𝘨}{\kappa }(v_{w}-v_{s})\\ F_{w}=n^{2}\frac{\rho_{w}𝘨}{\kappa }(v_{s}-v_{w}) \end{array}\right.$$

where *n* is the porosity of porous rock mass media, $\rho_{w}$ is the density of water, *g* is the acceleration of gravity, and $v_{w}$ is the average velocity of virtual water particles interacting with a given solid particle. Similarly, $v_{s}$ is the average velocity of solid particles interacting with a given virtual water particle.

According to the particle approximation method of SPH, $v_{w}$ and $v_{s}$ in Equation (16) can be obtained by discrete equations as follows:

(17) $$\left\{\begin{array}{l} v_{w,i}=\sum_{j}\frac{m_{j}}{\rho_{0j}}v_{w,j}W_{0ij}\\ v_{s,i}=\sum_{j}\frac{m_{j}}{\rho_{0j}}v_{s,j}W_{0ij} \end{array}\right.$$

where $W_{0ij}=W_{0}(|X_{i}-X_{j}|,h)$ is the kernel function in the initial configuration, $m_{j}$ is the mass of solid particle *j*, and $\rho_{0j}$ is the density of solid particle *j*.

Similarly, Equations (10) and (15) can be discretized into the following forms:

(18) $$\left\{\begin{array}{l} L_{i}=\sum_{j}\frac{m_{j}}{\rho_{0j}}(u_{j}-u_{i})\cdot \frac{\partial W_{0ij}}{\partial X_{i}}\\ F_{i}=\sum_{j}\frac{m_{j}}{\rho_{0j}}(x_{j}-x_{i})\cdot \frac{\partial W_{0ij}}{\partial X_{i}}\\ \frac{dv_{i}}{dt}=\sum_{j}m_{j}(\frac{P_{i}(x,p,t)}{\rho_{0i}^{2}}+\frac{P_{j}(x,p,t)}{\rho_{0j}^{2}}+P_{av,i})\frac{\partial W_{0ij}}{\partial X_{i}}+\sum_{j}m_{j}\frac{F_{s,i}}{\rho_{0}{}_{i}\rho_{0j}}W_{0ij} \end{array}\right.$$

where $F_{s,i}$ is the osmotic pressure acting on solid particle *i*, $P_{av,i}$ is the artificial viscosity force, and $P_{av,i}=\det(F_{i})\Pi_{\;ij}F_{i}^{-T}$.

In this paper, the linear and quadratic viscosity combination form proposed by Monaghan and Gingold [46] was adopted:

(19) $$\Pi_{\;ij}=\left\{\begin{array}{ll} \frac{-\alpha {\bar{c}}_{ij}u_{ij}+\beta u_{ij}^{2}}{{\bar{\rho }}_{ij}} & ,\;\;v_{ij}\cdot x_{ij}<\;0\\ 0 & ,\;\;v_{ij}\cdot x_{ij}\geq \;0 \end{array}\right.$$

where $u_{ij}=hv_{ij}x_{ij}/(x_{ij}^{2}+\varepsilon h^{2})$, and *h* is the smoothing length; *α* and *β* are numerical parameters related to artificial viscosity with a value of 1.0; *ε* is a constant with a value of 0.01; and ${\bar{\rho }}_{ij}=(\rho_{i}+\rho_{j})/2$ ${\bar{c}}_{ij}=(c_{i}+c_{j})/2$ are the mean value of the density and the sound velocity of two interacting particles, respectively, where the sound velocity $c=\sqrt{4G/3\rho_{0}}$. $v_{ij}=v_{i}-v_{j}$ and $x_{ij}=x_{i}-x_{j}$ are the relative velocity and relative position between two interacting particles, respectively.

### 3.3. Seepage Control Equation Considering Hydro-Mechanical Damage Coupling Effect

Yang [47] deduced the relationship between the permeability coefficient, principal stress, and pore water pressure according to the changes of pores:

(20) $$\kappa (\sigma ,p)=\kappa_{0}\exp\left[-a(\sigma_{kk}/3-\alpha p)/H^{\prime }\right]$$

where $\kappa_{0}$ is the initial permeability coefficient of porous rock mass medium, *a* is the coupling parameter, $\sigma_{kk}/3$ is the mean value of the principal stress, and $H^{\prime }$ is the Biot constant.

Let $\kappa_{s}=\kappa_{d}=\kappa_{0}$ in Equation (5), and substitute them into Equation (20); the equivalent continuous permeability coefficient of porous rock mass medium and the permeability coefficient of the macroscopic defect surface under the hydro-mechanical coupling condition can be obtained as follows:

(21) $$\left\{\begin{array}{l} \kappa_{s}(\sigma ,p)=\frac{𝘨\lambda b_{0}^{3}}{24u}\exp\left[-a(\sigma_{kk}/3-\alpha p)/H^{\prime }\right]\\ \kappa_{d}(\sigma ,p)=\frac{𝘨b_{0}^{2}}{12u}\exp\left[-a(\sigma_{kk}/3-\alpha p)/H^{\prime }\right] \end{array}\right.$$

Similar to Equation (7), the permeability coefficient of porous rock mass medium under the coupling condition of hydro-mechanical damage can be expressed as follows:

(22) $$\kappa_{t}(\sigma ,p)=\psi_{d}\kappa_{d}(\sigma ,p)+\psi_{s}\kappa_{s}(\sigma ,p)$$

Then the permeability coefficient of virtual link in porous rock mass media under the coupling condition of hydro-mechanical damage is

(23) $$\kappa_{t,ij}(\sigma ,p)=\frac{\kappa_{t,i}(\sigma ,p)+\kappa_{t,j}(\sigma ,p)}{2}$$

According to Equations (4), (6), and (21)–(23), the discrete formula of the seepage control equation under the coupled condition of hydro-mechanical damage can be obtained as follows:

(24) $$\left\{\begin{array}{l} \psi_{d,i}=\frac{D_{i}-c_{1}}{c_{2}-c_{1}},\;\psi_{s,i}=1-\psi_{d,i}\\ \kappa_{t,i}(\sigma ,p)=\;\frac{𝘨b_{0}^{2}(\lambda b_{0}\psi_{s,i}+2\psi_{d,i})}{24u}\exp\left[-a(\sigma_{kk,i}/3-\alpha p_{i})/H^{\prime }\right]\\ v_{w,i}=\frac{\kappa_{t,i}(\sigma ,p)+\kappa_{t,j}(\sigma ,p)}{2}\sum_{j}\frac{m_{j}}{\rho_{j}}(H_{i}-H_{j})\frac{\partial W_{ij}}{\partial x_{i}}\\ \frac{\partial H_{i}}{\partial t}=\frac{1}{S_{i}}\sum_{j}\frac{m_{j}}{\rho_{j}}(v_{w,i}-v_{w,j})\frac{\partial W_{ij}}{\partial x_{i}} \end{array}\right.$$

### 3.4. Failure Criteria and Damage Treatment

**Figure 11:**
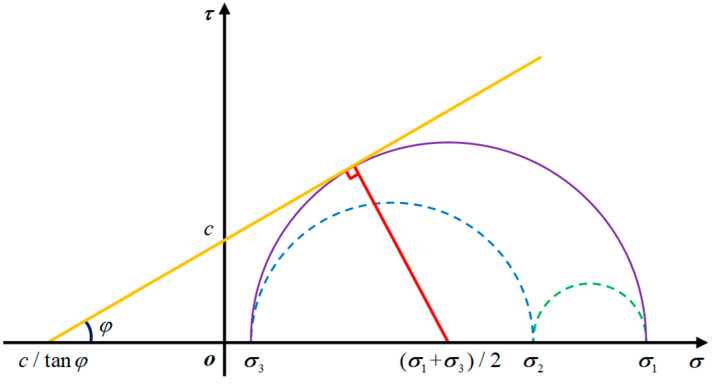
Mohr–Coulomb failure criterion, reprinted from Ref. [48].

Since rocks are mainly brittle materials, the application of an appropriate strength criterion is critical to accurately describe crack propagation modes in rocks. In this work, the Mohr–Coulomb failure criterion and maximum principal stress criterion were used to determine the different failure types of rocks, namely, shear failure and tension failure. When the maximum principal stress suffered by the virtual link exceeds the tensile strength of the material, the virtual link will undergo tensile failure. As shown in Figure 11, when the shear stress of the virtual link conformed to the Mohr–Coulomb failure criterion, the shear failure occurred on the virtual link. The Mohr–Coulomb failure criterion is defined as follows:

(25) $$\frac{1}{2}(\sigma_{1}-\sigma_{3})=\left[\frac{c}{\tan\varphi }+\frac{1}{2}(\sigma_{1}+\sigma_{3})\right]\sin\varphi$$

where $\sigma_{1}$ is the maximum principal stress; $\sigma_{3}$ is the minimum principal stress; and *c* and φ are the cohesion and internal friction angle, respectively.

The stress on the virtual link can be obtained by averaging the stress of two interacting particles:

(26) $$\sigma_{ij}=(\sigma_{i}+\sigma_{j})/2$$

**Figure 12:**
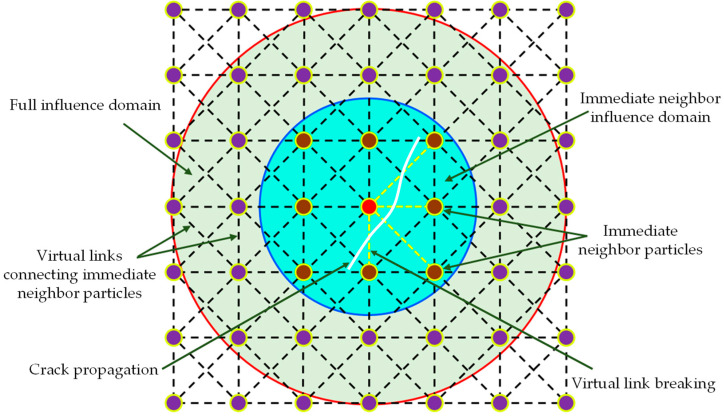
Virtual link fracture and crack propagation in the immediate neighborhood, adapted from Refs. [50,51].

In the SPH method, the bell-shaped kernel ensures that the interaction between particles is inversely proportional to their distance, and this property of the kernel inspires the hypothesis of damage treatment methods. In the present approach, the neighbor definition of particles was modified so that particle only interacted with immediate neighbor particles (see Figure 12). In order to reflect the interaction between adjacent particles, a numerical parameter *f_ij_* called the “interaction factor” by Chakraborty and Shaw [49] was embedded into the numerical model, whose value depends on the damage state of the virtual link. Initially, unbroken virtual links meant “full interaction” with *f_ij_* = 1, while broken virtual links meant “no interaction” with *f_ij_* = 0. When the virtual link was damaged by external forces, the interaction between the particles no longer occurred, and the microcrack gradually started from the broken virtual link, as shown in Figure 12.

Therefore, after introducing interaction factor *f_ij_*, Equation (18) can be re-expressed as follows:

(27) $$\left\{\begin{array}{l} {\tilde{L}}_{i}=\sum_{j}f_{ij}\cdot \frac{m_{j}}{\rho_{0,j}}(u_{j}-u_{i})\cdot \frac{\partial W_{0ij}}{\partial X_{i}}\\ {\tilde{F}}_{i}=\sum_{j}f_{ij}\cdot \frac{m_{j}}{\rho_{0,j}}(x_{j}-x_{i})\cdot \frac{\partial W_{0ij}}{\partial X_{i}}\\ \frac{d{\tilde{v}}_{i}}{dt}=\sum_{j}f_{ij}\cdot m_{j}(\frac{P_{i}(x,p,t)}{\rho_{0i}^{2}}+\frac{P_{j}(x,p,t)}{\rho_{0j}^{2}}+P_{av,i})\frac{\partial W_{0ij}}{\partial x_{i}}+\sum_{j}m_{j}\frac{F_{s,i}}{\rho_{0}{}_{i}\rho_{0j}}W_{0ij} \end{array}\right.$$

**Figure 13:**
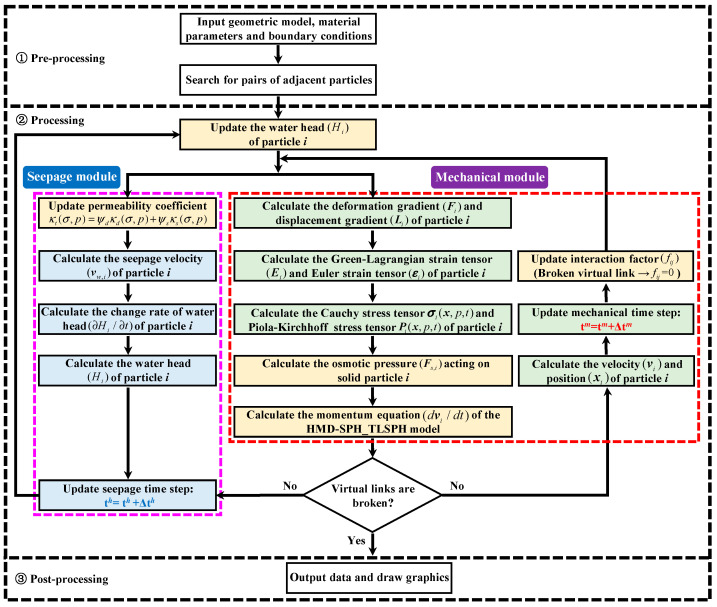
The program calculation flow chart of the EHM-TLF-SPH model considering osmotic pressure.

The hydro-mechanical coupling program included a seepage module and a mechanical module. For the seepage module, the permeability coefficient of the particle considering the mechanical damage coupling effect was first calculated according to Equation (24), and then the permeability coefficient of the virtual link was obtained. Second, according to Equation (9), the seepage velocity of particles and the change rate of water head were obtained. Then, the change rate of water head was leapfrog integrated to update the water head of particles. For the mechanical module, the Green–Lagrange strain tensor of the particle was calculated according to Equations (10)–(12). Second, the Cauchy stress tensor of the particle was obtained by substituting the water pressure into the stress–strain constitutive relation shown in Equation (13), and then the first kind of Piola–Kirchhoff stress tensor was obtained by Equation (14). Meanwhile, according to Equation (16), the osmotic pressure acting on solid particles and osmotic reaction acting on virtual water particles could be obtained. Finally, the acceleration of particles considering osmotic pressure and hydrostatic pressure could be calculated by the momentum equation in Equation (18) to update the particle’s velocity and position. Once the stress on the virtual link met the strength criterion, the virtual link broke, the interaction factor *f_ij_* changed from 1 to 0, and the seepage channel between the interacting particles gradually increased. In addition, the effective stress of the particles affected the permeability coefficient of the seepage domain. Therefore, according to Equation (24), the permeability coefficient of the virtual connection was updated, and the seepage velocity and the change rate of the head were calculated. Then, the stress–seepage coupling was calculated for the next cycle. In order to more clearly show the specific implementation process of the calculation program, the program calculation flow chart of the EHM-TLF-SPH model considering osmotic pressure is shown in Figure 13.

## 4. Simulation Results and Analysis

The osmotic pressure of the flaw water on the fractured surface determined the crack propagation and coalescence mode of the rock sample with pre-existing flaws. The osmotic pressure was significantly affected by the permeability coefficient and the flaw water pressure. Therefore, the rock sample with a single pre-existing flaw was first simulated to determine the effect of the permeability coefficient and flaw water pressure on the osmotic pressure. Then, the numerical simulation of uniaxial compression was carried out on the rock samples with pre-existing cracks to reveal the crack initiation and propagation mechanism of the fractured rock mass under different flaw water pressures.

### 4.1. Influence of Permeability Coefficient and Flaw Water Pressure on Osmotic Pressure

**Figure 14:**
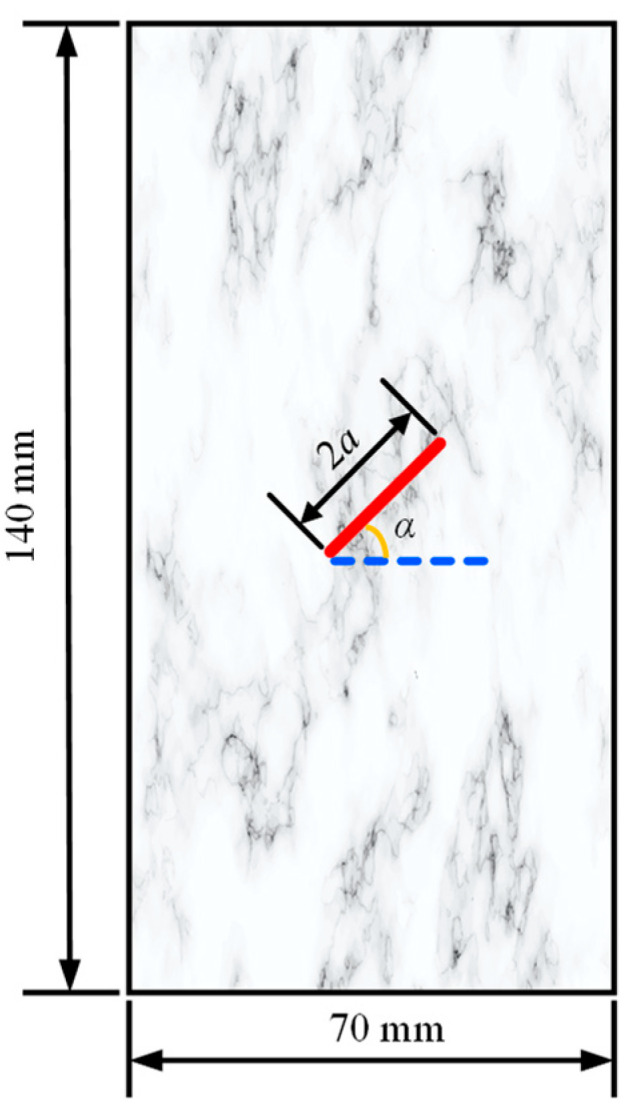
Geometric model of a rock sample with a single prefabricated flaw.

In order to investigate the effect of the flaw water pressure and the permeability coefficient on the osmotic pressure, a numerical rock sample of 140 mm × 70 mm in size was selected for seepage tests under different permeability coefficients and flaw water pressures. As shown in Figure 14, the inclination angle of the prefabricated flaw was 45°, and its length and width were 20 mm and 2 mm, respectively.

The numerical model was represented by 17,672 particles, and the distance between adjacent particles was Δ*x* = 0.75 m. The seepage integration step was Δt*^h^* = 1.0 × 10^−7^ s, and the influence domain radius was *δ* = 3.015d*x*. The numerical parameters of the rock sample were basically consistent with the experiments of Wei et al. [52], as shown in Table 3.

**Figure 15:**
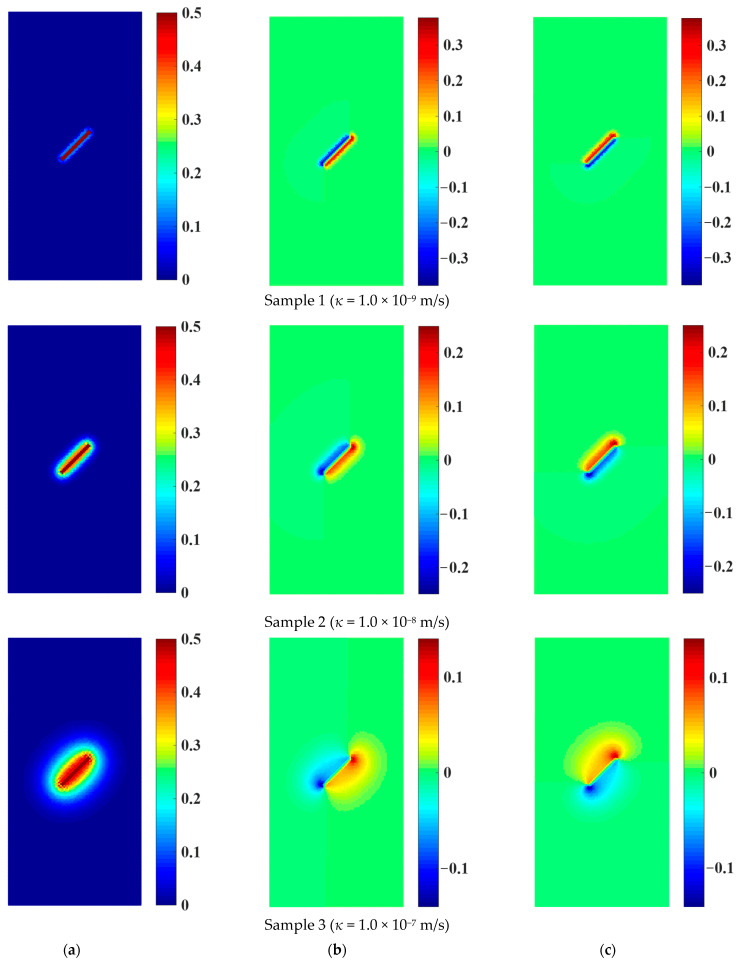
The pore water pressure and osmotic pressure of samples with a prefabricated flaw under different permeability coefficients (*p* = 0.5 MPa). (**a**) Pore water pressure. (**b**) Horizontal osmotic pressure. (**c**) Vertical osmotic pressure.

Figure 15 shows the distribution of the pore water pressure and osmotic pressure of rock samples containing a prefabricated flaw with a water pressure of 0.5 MPa under different permeability coefficients. As shown in Figure 15a, with the permeability coefficient increased, the seepage velocity and diffusion range of flaw water gradually increased. The pore water pressure decreased uniformly from inside to outside in the direction parallel to the prefabricated flaw, and the pore water pressure on the prefabricated flaw surface was the largest. The direction of the pore water pressure was perpendicular to the prefabricated flaw surface, promoting the tensile failure of the prefabricated flaw. Since the pore water pressure at the tip and surface of the prefabricated flaw was larger than that at the other positions, the pore water velocity herein was relatively large driven by the pressure gradient. Therefore, the osmotic pressure at the tip of the prefabricated flaw was the largest, followed by that on the flaw surface, as shown in Figure 15b,c. However, the osmotic pressure of the flaw water acting on the rock particles was determined by the permeability coefficient of the porous rock mass media and the seepage velocity of the pore water. With the increase in the permeability coefficient, although the seepage velocity of the pore water increased gradually, the hydraulic gradient of the pore water decreased faster. Therefore, with the passage of time, the larger the permeability coefficient of the rock sample, the smaller the osmotic pressure of the pore water acting on the solid particles, as shown in Figure 15b,c.

**Figure 16:**
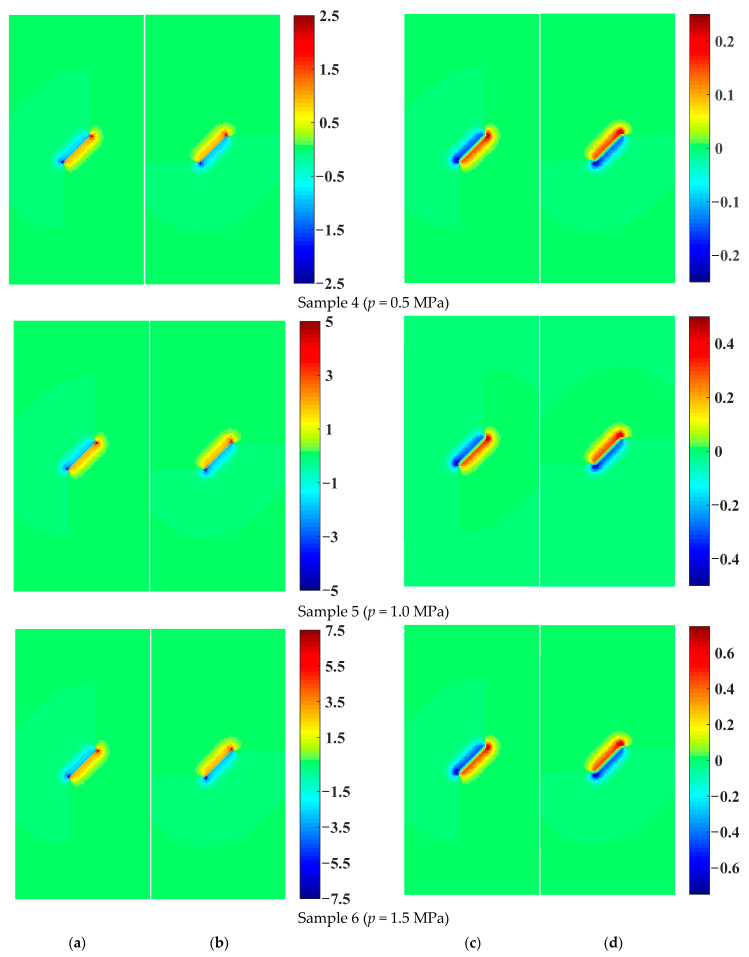
The seepage velocity and osmotic pressure distribution of rock samples with a prefabricated flaw under different flaw water pressures (*κ* = 1.0 × 10^−8^ m/s). (**a**) Horizontal seepage velocity (unit: ×10^−4^ m/s). (**b**) Vertical seepage velocity (unit: ×10^−4^ m/s). (**c**) Horizontal osmotic pressure (unit: MPa). (**d**) Vertical osmotic pressure (unit: MPa).

When the permeability coefficient *κ* = 1.0 × 10^−8^ m/s, the seepage velocity and osmotic pressure distribution of rock samples with a prefabricated flaw under different flaw water pressures are shown in Figure 16. It can be seen from Figure 16a,b that with the increase in flaw water pressure, the seepage velocity of the pore water increased exponentially. When the permeability coefficient was constant, the osmotic pressure was only related to the seepage velocity. Therefore, contrary to the distribution of the osmotic pressure in Figure 16, the seepage pressure acting on the rock particles increased exponentially with the increase in the flaw water pressure. As shown in Figure 16, when the flaw water pressure increased by three times, that is, from 0.5 MPa to 1.5 MPa, the maximum osmotic pressure of the tip and surface of the prefabricated flaw increased by about three times. When the permeability coefficient increased by 100 times, that is, from 1.0 × 10^−9^ m/s to 1.0 × 10^−7^ m/s, the maximum osmotic pressure of the tip and surface of the prefabricated flaw decreased by about three times (see Figure 16). It could be seen that the influence of the flaw water pressure on the osmotic pressure of the tip and surface of the prefabricated flaw was much greater than that on the permeability coefficient. Therefore, the larger the flaw water pressure, the smaller the permeability coefficient, and the larger the osmotic pressure of the tip and surface of the prefabricated flaw, the easier the tip of the prefabricated flaw is to crack. However, if the permeability coefficient was too small, the seepage velocity and diffusion range were seriously affected, which was not conducive to the propagation and coalescence of cracks. In hydraulic cracking engineering, in order to achieve a better cracking effect, the permeability coefficient can be controlled in a reasonable range by temperature control, and then the flaw water pressure could be increased to crack. The numerical results showed that the EHM-TLF-SPH model could well reveal the hydraulic cracking mechanism and had high engineering application value.

### 4.2. Crack Initiation and Propagation of Rock Samples with a Prefabricated Flaw under Uniaxial Compression and Flaw Water Pressure

In order to demonstrate the validity of the EHM-TLF-SPH model and reveal the seepage fracture mechanism of rock samples with a prefabricated flaw, rock samples with the same geometric size and flaw morphology as Section 4.1 were selected for numerical simulation under uniaxial compression and flaw water pressure. The numerical model was represented by 17,672 particles, and the distance between adjacent particles was Δ*x* = 0.75 m. The seepage integration step was Δt*^h^* = 1.0 × 10^−7^ s, the mechanical integration step was Δt*^m^* = 1.0 × 10^−7^ s, and the influence domain radius was *δ* = 3.015d*x*. Moreover, the top and bottom of the model were simultaneously subjected to displacement loading at a speed of 0.5 m/s. The mechanical parameters of the rock sample with a prefabricated flaw are shown in Table 3.

In order to make the numerical simulation more consistent with the laboratory experiment, the tensile strength and cohesive of model particles follow the Weibull distribution to describe the non-uniform characteristics of real rock samples. The specific form of the Weibull distribution function [53] is as follows:

(28) $$W(x)=\frac{\omega }{\lambda_{0}}{\left(\frac{\lambda }{\lambda_{0}}\right)}^{\omega -1}\exp\left[-{\left(\frac{\lambda }{\lambda_{0}}\right)}^{\omega }\right]$$

where ω is the homogeneity index with a value of 10 in this section, λ is the mechanical parameter, and $\lambda_{0}$ is the mean value of the mechanical parameters.

**Figure 17:**
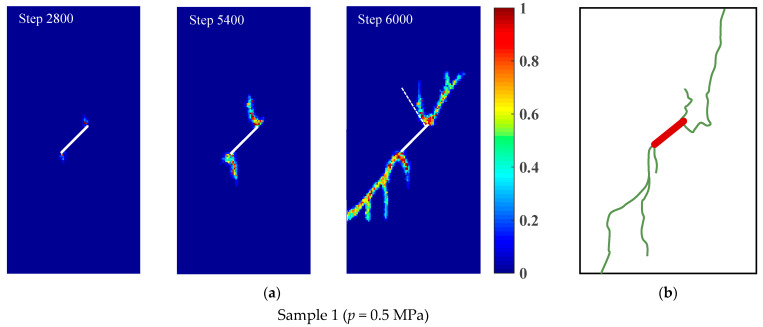

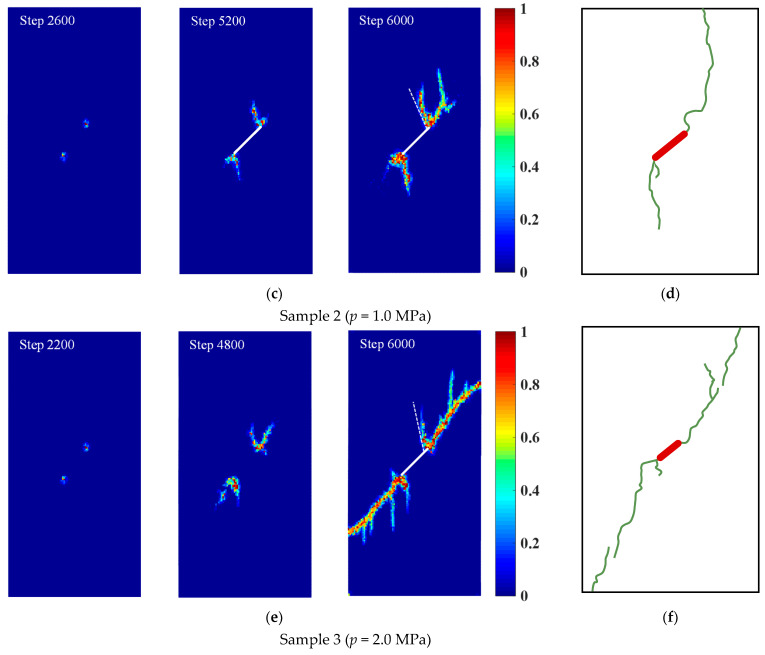
Crack initiation and propagation of rock samples with a prefabricated flaw under uniaxial compression and different flaw water pressures. (**a**,**c**,**e**) Numerical results. (**b**,**d**,**f**) Experimental result, reprinted from Ref. [52].

The crack initiation and propagation of rock samples with a prefabricated flaw under uniaxial compression and different flaw water pressures is shown in Figure 17. For rock sample 1, when the loading step was 2800, the wing crack first emanated from the tip of the prefabricated flaw. When the loading step was 5400, the wing crack gradually propagated to both ends of the rock sample in the direction of the maximum principal stress. Meanwhile, the quasi-coplanar shear crack started from the tip of the prefabricated flaw. When the loading step was 6000, the wing crack initiated by the prefabricated flaw no longer grew. The quasi-coplanar shear crack that started from the tip of the prefabricated flaw propagated rapidly and penetrated the left edge of the model, and then failure occurred in rock sample 1. For rock sample 2, when the loading step was 2600, the wing crack first began from the tip of the prefabricated flaw. When the loading step was 5200, the quasi-coplanar shear crack emanated from the tip of the prefabricated flaw. When the loading step was 6000, the wing crack that started from the prefabricated flaw continued to propagate. As shown in Figure 17c, the horsetail crack started from the quasi-coplanar shear crack initiated from the tip of the prefabricated flaw and propagated rapidly, and then failure occurred in rock sample 2. For rock sample 3, when the loading step was 2200, the wing crack first emanated from the tip of the prefabricated flaw. When the loading step was 4800, the quasi-coplanar shear crack generated from the tip of the prefabricated flaw. When the loading step was 6000, the wing crack initiated by the prefabricated flaw no longer propagated, while the quasi-coplanar shear that started from the tip of the prefabricated flaw propagated rapidly and penetrated the left and right edges of the model. Then, the failure occurred in rock sample 3. In addition, the crack propagation modes of rock samples 1–3 were compared with the experimental results by Wei et al. [52], and the numerical results agreed well with the test results, as shown in Figure 17b,d,f.

**Figure 18:**
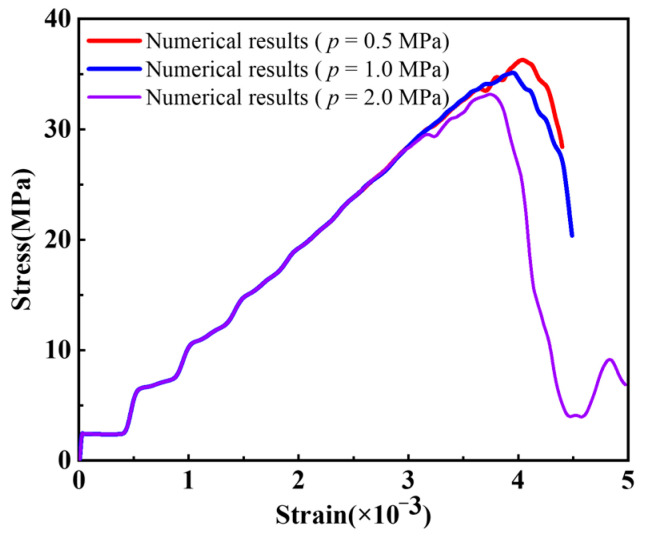
Stress−strain curves of rock samples with a prefabricated flaw under uniaxial compression and different flaw water pressures.

Figure 18 shows the stress–strain curves of rock samples with a prefabricated flaw under uniaxial compression and different flaw water pressures. The numerical results in Section 4.1 showed that for rock materials with low permeability, less flaw water infiltrated into the porous rock mass media from the prefabricated flaw, and the hydrostatic pressure generated by the flaw water had little effect on the strength of the sample. Therefore, the stress–strain curves of the rock samples containing a prefabricated flaw with different water pressures at the elastic stage were basically consistent (see Figure 18). In addition, it could be seen from Figure 18 that as the flaw water pressure increased, the compressive strength and peak strain of the rock sample decreased gradually. The compressive strengths of the rock samples containing a prefabricated flaw with water pressures of 1.0 MPa and 2.0 MPa were 5.52% and 9.36% lower, respectively, than that of the rock sample containing a prefabricated flaw with a water pressure of 0.5 MPa. The peak strains of rock samples containing a prefabricated flaw with water pressures of 1.0 MPa and 2.0 MPa were 6.18% and 7.90% lower, respectively, than that of the rock sample containing a prefabricated flaw with a water pressure of 0.5 MPa. It could be seen that the osmotic pressure acting on the rock particles had a great influence on the strength characteristics of low-permeability rock samples with prefabricated flaws, which greatly accelerated the fracture process of the rock samples.

**Figure 19:**
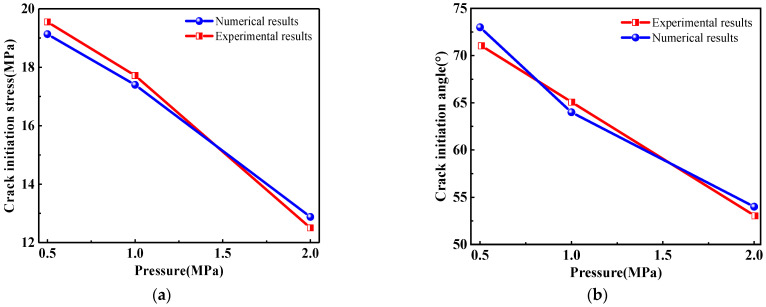
Crack initiation stress and crack initiation angle of rock samples with a prefabricated flaw under uniaxial compression and different flaw water pressures. (**a**) Crack initiation stress. (**b**) Crack initiation angle.

Figure 19 shows the crack initiation stress and initiation angle of rock samples with a prefabricated flaw under uniaxial compression and different flaw water pressures. It could be seen from Figure 19a that as the flaw water pressure increased, the crack initiation stress gradually decreased. Due to the increase in the flaw water pressure, the osmotic pressure acting on the tip and surface of the prefabricated flaw increased obviously, which changed the maximum principal stress field and the maximum shear stress field at the tip of the prefabricated flaw. In addition, the increase in the flaw water pressure intensified the splitting effect of flaw water on the tip and surface of the prefabricated flaw, which not only accelerated the initiation of tension cracks but also increased the shear driving force of compression shear cracks, resulting in the continuous propagation of shear cracks.

Figure 19b shows that as the flaw water pressure increased, the crack initiation angle of the sample gradually decreased. This was due to the fact that the flaw water reduced the lateral pressure and friction coefficient between the upper and lower flaw surfaces. The numerical results agreed well with the research results of Shen [54] on the influence of lateral pressure and friction coefficient on crack propagation. Furthermore, Figure 19 shows that the maximum relative errors of the crack initiation stress and crack initiation angle were 3.0% and 2.8%, respectively. The numerical results agreed well with the previous experimental observations [52], verifying the validity of the EHM-TLF-SPH model.

### 4.3. Crack Propagation and Coalescence of Rock Samples with Two Prefabricated Flaws under Uniaxial Compression and Flaw Water Pressure

**Figure 20:**
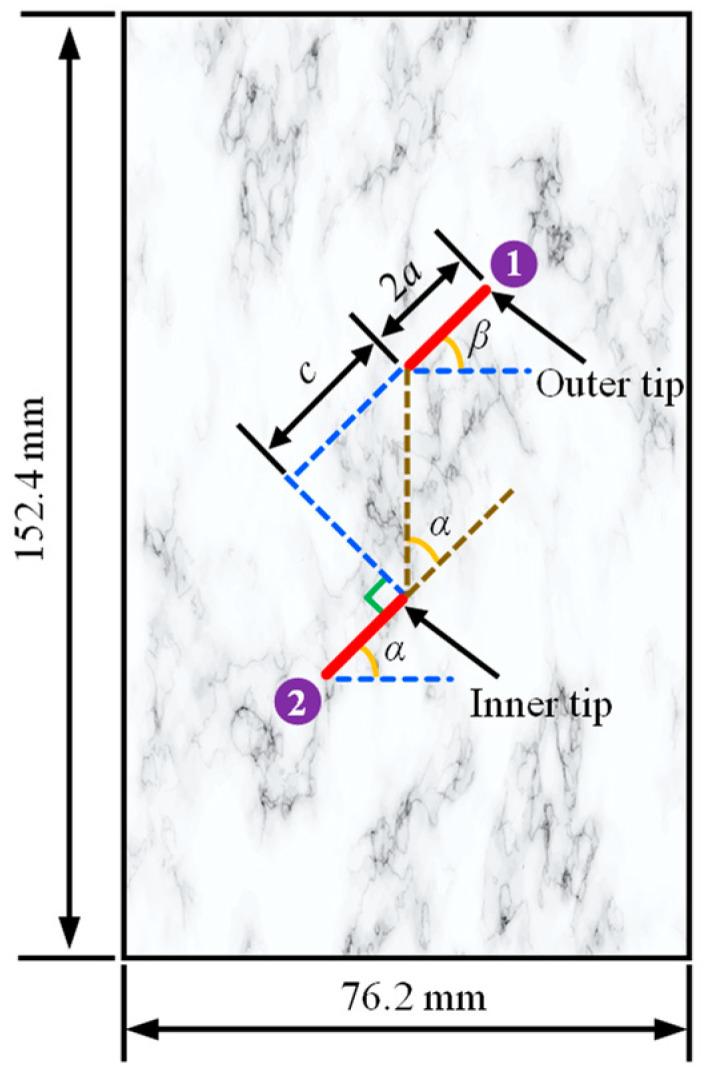
Geometric model of the sample with two parallel prefabricated flaws.

In order to investigate the effect of flaw water pressure on crack propagation and coalescence mode of rock samples with two parallel prefabricated flaws, three samples with different flaw water pressures were selected for numerical simulation. As shown in Figure 20, the size of the numerical sample was 152.4 mm × 76.2 mm, and the inclination angle of the prefabricated flaw was *β* = 45°. The angle between the connecting line between the inner tips of the prefabricated flaws 1 and 2 and the extension line of the prefabricated flaw 2 was *α* = 45 °. The numerical model was represented by 20,503 particles, and the distance between adjacent particles was Δ*x* = 0.75 m. The mechanical time step was Δt*^m^* = 5.0 × 10^−8^ s, and the influence domain radius was *δ* = 3.015d*x*. Besides, the top and bottom of the model were loaded with displacement at a loading speed of 0.5 m/s. The mechanical parameters of the sample with two parallel prefabricated flaws were shown in Table 4.

**Figure 21:**
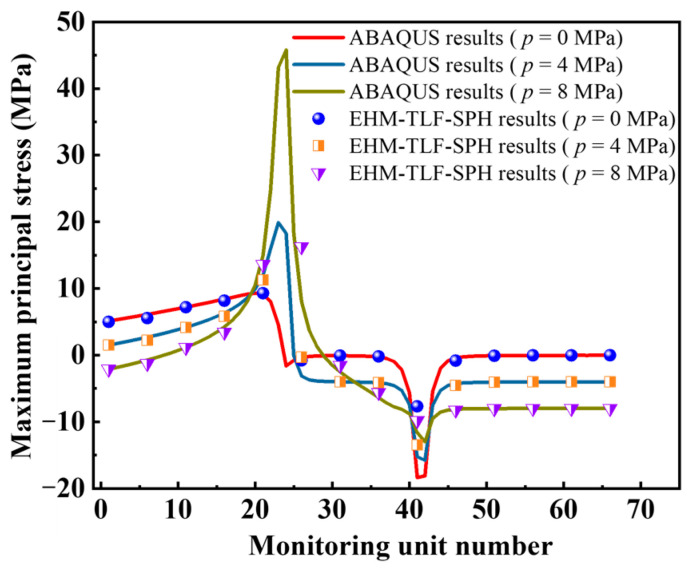
Maximum principal stress at monitoring points of rock samples under uniaxial compression and different flaw water pressures.

**Figure 22:**
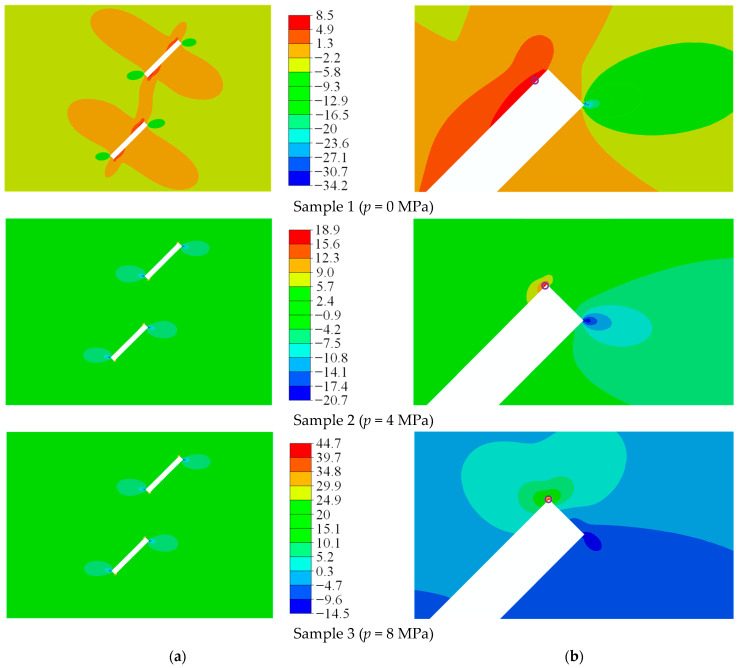
Maximum principal stress distribution of rock samples with two parallel prefabricated flaws under uniaxial compression and different flaw water pressures (ABAQUS results). (**a**) Maximum principal stress. (**b**) Detailed view of the inner tip of the prefabricated flaw 2.

In the numerical model, the inner tip of the prefabricated flaw 2 was evenly distributed with 14 monitoring particles. In order to more clearly observe the distribution characteristics of the maximum principal stress at the inner tip of the prefabricated flaw 2, the 14 monitoring particles were divided into 66 monitoring units in ABAQUS. Then, the present numerical results were compared with the ABAQUS results. For the convenience of description, the three samples were numbered as sample 1 (*p* = 0 MPa), sample 2 (*p* = 4 MPa), and sample 3 (*p* = 8 MPa). Figure 21 shows the maximum principal stress at monitoring points of rock samples under uniaxial compression and different flaw water pressures. As can be seen from Figure 21, a peak and a trough were observed in the maximum principal stress curves of samples 2 and 3. In order to more clearly observe the change of the concentration position of the maximum principal stress at the tip of the prefabricated flaw 2 of samples 1–3, the maximum principal stress distribution of rock samples with two parallel prefabricated flaws under uniaxial compression and different flaw water pressures is shown in Figure 22. It can be seen from the ABAQUS results that the wave crest and wave trough were located at the upper left and lower right corners, respectively, of the inner tip of the prefabricated flaw 2 (see Figure 22b). Since the concentrated position of the maximum principal stress of sample 1 was located in the front and lower right corner of the inner tip of the prefabricated flaw 2, only a wave trough was observed on the maximum principal stress curve of sample 1. Meanwhile, it could be found that the maximum principal tensile stress and maximum principal compressive stress of the inner tip of the prefabricated flaw 2 were located on the upper and lower surfaces of the prefabricated flaw 2, respectively.

**Figure 23:**
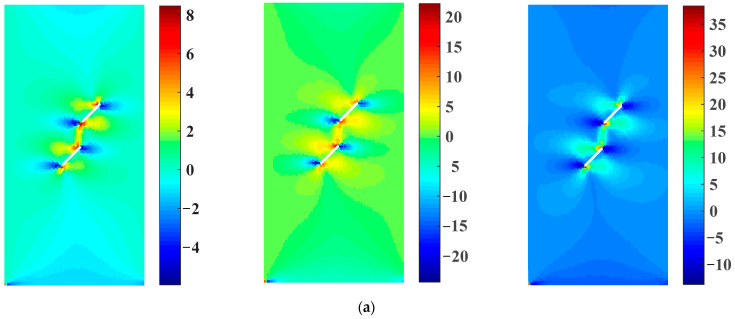

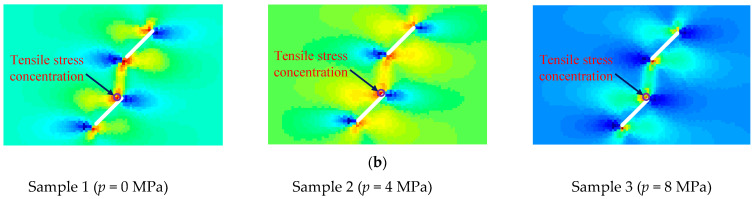
Maximum principal stress distribution of rock samples with two parallel prefabricated flaws under uniaxial compression and different flaw water pressures (EHM-TLF-SPH results). (**a**) Maximum principal stress. (**b**) Detailed view of the inner tip of the prefabricated flaw 2.

Figure 23 shows the maximum principal stress distribution of the rock samples with two parallel prefabricated flaws under uniaxial compression and different flaw water pressures obtained from the EHM-TLF-SPH model. It can be seen from Figure 23b that with the increase in the flaw pressure, the concentration position of the maximum principal stress at the inner tip of the prefabricated flaw 2 gradually shifted to the flaw tip, which was consistent with the present ABAQUS numerical observations (see Figure 22b) and the research conclusions of Silva and Einstein [2]. Meanwhile, it could be seen from Figure 23a that the peak values of the maximum principal stress of samples 1–3 obtained from the current numerical model were 8.63 MPa, 18.52 MPa, and 35.40 MPa, respectively. As shown in Figure 22a, the peak values of the maximum principal stress of samples 1–3 obtained by ABAQUS were 8.45 MPa, 18.90 MPa, and 44.70 MPa, respectively. For samples 1 and 2, the numerical results obtained by the EHM-TLF-SPH model agreed well with those obtained by ABAQUS, while for sample 3, there was some error between the present numerical results and the ABAQUS results. Since the maximum principal stress of sample 3 was concentrated in the upper left corner of the inner tip of the prefabricated flaw 2, the numerical error was caused by the shape effect, as shown in Figure 22b. In addition, for the same monitoring position, the maximum principal stress of the monitored particles obtained by the EHM-TLF-SPH model was in good agreement with that of the monitoring units obtained by ABAQUS (see Figure 21), further verifying the accuracy of the EHM-TLF-SPH model.

**Figure 24:**
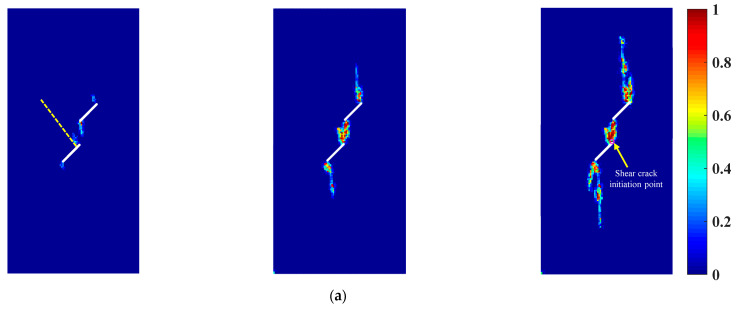

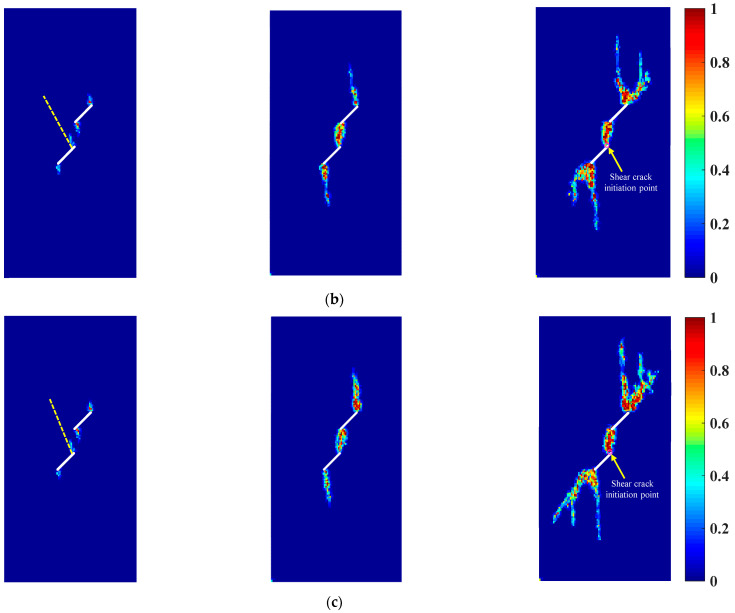
Crack propagation and coalescence modes of the sample with two parallel prefabricated flaws under uniaxial compression and different flaw water pressures (*α* = 45°, *β* = 45°). (**a**) Sample 1 (*p* = 0 MPa). (**b**) Sample 2 (*p* = 4 MPa). (**c**) Sample 3 (*p* = 8 MPa).

Figure 24 shows the crack propagation and coalescence modes of the sample with two parallel prefabricated flaws under uniaxial compression and different flaw water pressures. It could be seen from Figure 24 that with the increase in the flaw water pressure, the crack initiation position of the wing crack initiated from the inner tip of the prefabricated flaw 2 gradually moved forward to the flaw tip, which was consistent with the previous research results. For sample 1, the inner tips of prefabricated flaws 1 and 2 were initially the initiation and growth of the wing crack. As the loading progressed, the shear crack started and propagated from the inner tip of the prefabricated flaw 2. Finally, the wing crack emanating from the inner tip of the prefabricated flaw 1 coalesced with the shear crack emanating from the inner tip of the prefabricated flaw 2, and then failure occurred in sample 1. For samples 2 and 3, the inner tips of the prefabricated cracks 1 and 2 were initially the initiation and growth of the wing crack. However, the wing cracks initiating from the inner tips of the prefabricated flaws 1 and 2 were significantly suppressed as the loading progressed. Finally, the shear crack initiated from the inner tip of the prefabricated flaw 2 coalesced with the inner tip of the prefabricated flaw 1, and then failure occurred in samples 2 and 3. In addition, it can be seen from Figure 24 that with the increase in the flaw water pressure, the shear crack initiation point gradually moved away from the inner tip of the prefabricated flaw 2, which was in good agreement with the previous numerical observation by Silva and Einstein [2].

**Figure 25:**
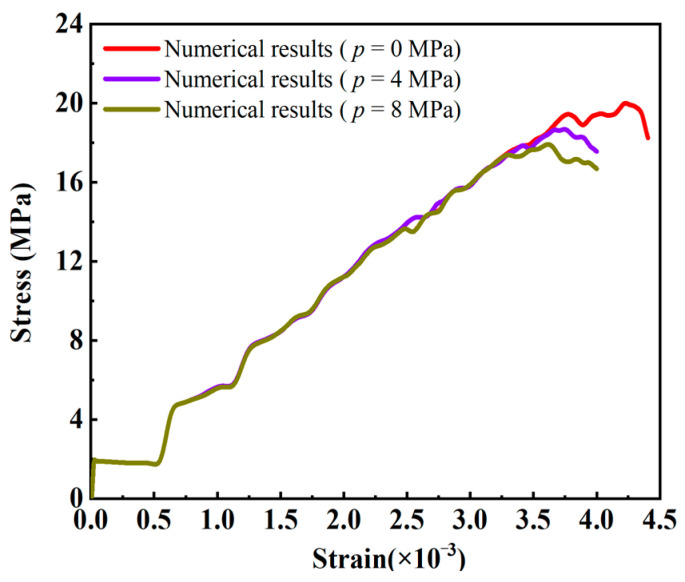
Stress–strain curves of rock samples with two parallel prefabricated flaws with different water pressures under uniaxial compression load.

Figure 25 shows the stress–strain curves of the rock samples with two parallel prefabricated flaws with different water pressures under uniaxial compression load. It can be seen from Figure 25 that the flaw water pressure had little effect on the stress–strain curve at the elastic stage of the rock sample with low permeability. In the damage deformation stage of the rock samples, the peak strain and peak strength of the rock samples gradually decreased with the increase in the flaw water pressure, as shown in Figure 25. Due to the low permeability of the rock samples, the osmotic pressure was the main driving force for the initiation and growth of the prefabricated flaw. Therefore, the larger the flaw water pressure, the more easily the rock sample cracks, and the smaller the peak strain and peak strength are. In addition, the strain softening characteristics of the rock samples could be observed from the stress–strain curve, that is, after reaching the peak stress, the stress–strain curve dropped to a low stress level.

In order to study the crack coalescence mode and seepage distribution of rock samples with high permeability containing two parallel prefabricated flaws, a rock-like material with the geometric size shown in Figure 20 was used for the uniaxial compression numerical simulation. In the present model, the inclination angles *α* and *β* were 30° and 45°, respectively; the flaw water pressure was *p* = 0.5 MPa; and the permeability coefficient of the sample was *κ* = 1.0 × 10^−7^ m/s. The numerical model was represented by 20,503 particles, and the distance between adjacent particles was Δ*x* = 0.75 m. The mechanical integration step was Δt*^m^* = 2.0 × 10^−7^ s, and the seepage time step was Δt*^h^* = 1.0 × 10^−6^ s. The influence domain radius was *δ* = 3.015d*x*, and the top of the model was loaded with downward displacement at a loading speed of 0.5 m/s. The mechanical parameters of a rock sample with two parallel prefabricated flaws are listed in Table 5.

**Figure 26:**
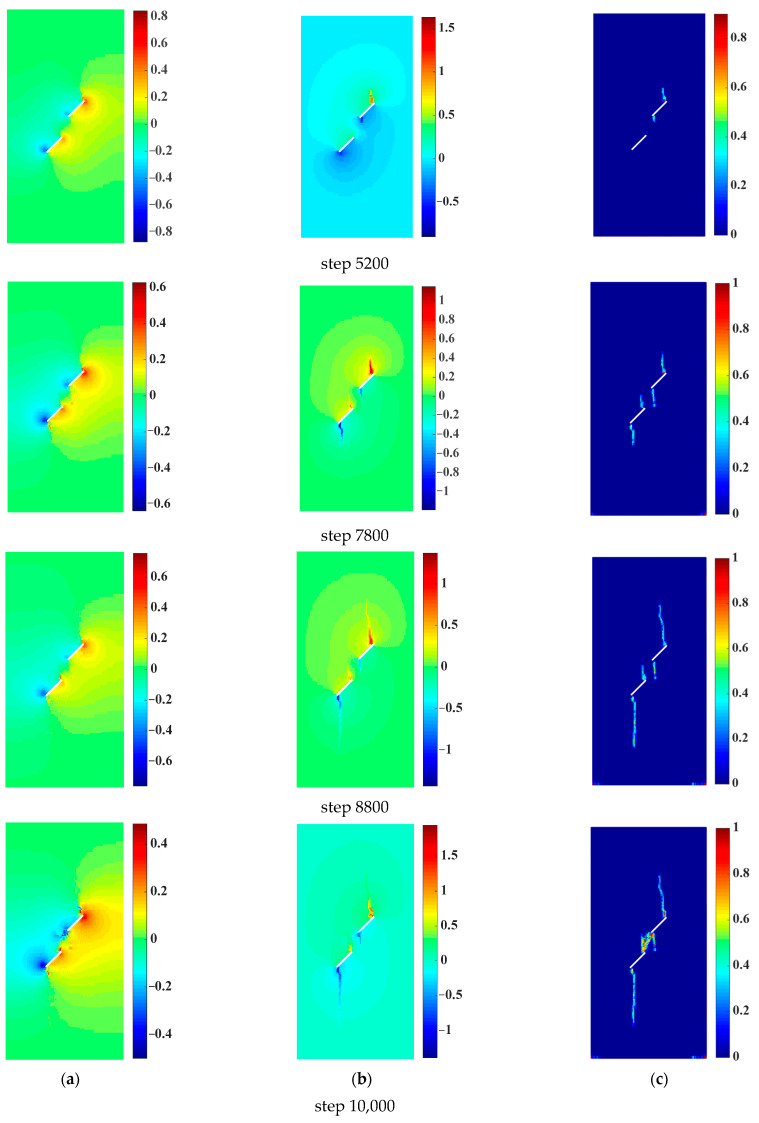
Crack propagation and coalescence modes of the sample with two parallel prefabricated flaws under uniaxial compression and 0.5 MPa flaw water pressure (*α* = 30°, *β* = 45°). (**a**) Horizontal seepage velocity (unit: mm/s). (**b**) Horizontal seepage velocity (unit: mm/s). (**c**) Crack propagation and coalescence.

Figure 26 shows the crack propagation and coalescence modes of the sample with two parallel prefabricated flaws under uniaxial compression and 0.5 MPa flaw water pressure. When the loading step was 5200, the wing crack initiated from the two tips of the prefabricated flaw 1 first, and the crack initiation velocity of the outer tip was faster than that of the inner tip. Then, the flaw water began to appear as obvious seepage at the outer tip of the prefabricated flaw 1, as shown in Figure 26b. When the loading step was 7800, the wing crack initiated from the two tips of the prefabricated flaws 1 and 2 propagated to both ends of the sample in the direction of the maximum principal stress. The flaw water continued to spread in the wing crack, and the seepage velocity of the flaw water decreased gradually. When the loading step was 8800, the wing cracks initiated by the outer tips of the prefabricated flaws 1 and 2 rapidly propagated to both ends of the rock sample. When the loading step was 10,000, the anti-plane shear crack emanated from the inner tip of the prefabricated flaw 2 coalesced with the inner tip of the prefabricated flaw 1, as shown in Figure 26c. The wing cracks starting from the outer tips of the prefabricated flaws 1 and 2 no longer propagated. Meanwhile, the vertical seepage velocity of the particles near the outer tip of the prefabricated flaw 1 increased sporadically, which was caused by the rapid damage of the particles at the outer tip of the prefabricated flaw 1 and unstable seepage of the flaw water. Then, failure occurred in the sample. As can be seen from Figure 26a,b, when the flaw water pressure was constant, the seepage velocity of the flaw water reached the maximum at the crack initiation stage. As the crack propagated, the hydraulic gradient gradually decreased, and the seepage velocity of the flaw water gradually decreased. It could be seen that the simulation results fully accorded with the seepage law of the crack propagation test under uniaxial compression.

**Figure 27:**
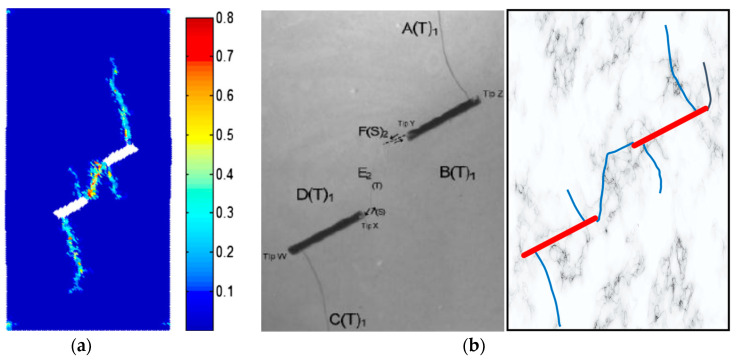
Crack propagation and coalescence of the sample containing two parallel prefabricated flaws with an inclination angle of 30° under uniaxial compression (*α* = 30°, *β* = 30°, and *p* = 0.5 MPa). (**a**) GPD results, reprinted from Ref. [4]. (**b**) Experimental results, reprinted from Ref. [55].

Figure 27 shows the crack propagation and coalescence modes of the sample containing two parallel prefabricated flaws with the inclination angles *α* = 30° and *β* = 30° obtained by GPD and experiments, respectively. The current numerical results agreed well with the GPD and test results, further verifying the accuracy and validity of the EHM-TLF-SPH model, as shown in Figure 26c and Figure 27.

## 5. Conclusions

In this paper, the seepage model based on the SPH method was developed and successfully applied to the seepage simulation of the two-dimensional five-spot well network. The simulation results agreed well with the analytical solutions, verifying the accuracy and robustness of the seepage model. Then, the linear interpolation function of phase field theory was introduced to establish the seepage model of porous rock mass media under the coupling condition of hydro-mechanical damage.

Then, a hydro-mechanical damage coupling model considering the osmotic pressure effect was proposed under the framework of TLSPH, successfully simulating the crack propagation and coalescence process of rock samples with prefabricated flaws under uniaxial compression and flaw water pressure. The results show that the crack initiation angle and stress of wing crack decreased gradually with the increase in the flaw water pressure. Meanwhile, it was observed that the compressive strength of the rock samples containing a prefabricated flaw with water pressures of 1.0 MPa and 2.0 MPa was 5.52% and 9.36% lower, respectively, than that of rock samples containing a prefabricated flaw with a water pressure of 0.5 MPa. Moreover, the maximum relative errors of the crack initiation angle and stress obtained by the simulation and the experimental results were 3.0% and 2.8%, respectively, verifying the accuracy of the EHM-TLF-SPH model.

In addition, the influence of the permeability coefficient and flaw water pressure on the osmotic pressure was also investigated. The numerical results showed that the larger the flaw water pressure, the smaller the permeability coefficient of porous rock mass media, the greater the osmotic pressure of the tip and surface of the prefabricated flaw, and the easier the tip of the prefabricated flaw was to crack. Therefore, the extended hydro-mechanical damage coupling model can well reveal the hydraulic fracturing mechanism and provide important technical support for hydraulic fracturing engineering involved in the mining of underground energy sources, such as geothermal and coal.

In this paper, the crack growth and coalescence processes of rock samples with pre-existing cracks under uniaxial compression and flaw water pressure were studied. However, the evolution process of cracks in 3D rock samples is usually much more complicated than that in 2D rock samples. Therefore, the propagation and coalescence of 3D cracks under complex loads and flaw water pressure need further investigation. In addition, the rock samples contain a variety of interlayers, whose mechanical properties are quite different. Therefore, the proposed coupled dynamic buffer SBT algorithm needs further improvement to deal with the stratified boundary between different lithologies.

## Figures and Tables

**Table 1:** The 2D seepage simulation parameters of an intact rock sample.

Inflow Water Pressure, *P*_I_ (MPa)	Outflow Water Pressure, *P*_II_ (MPa)	Permeability Coefficient, *κ*(m/d)	Compressibility of Rock Mass, *α* (1/Pa)	Compressibility of Water, *β* (1/Pa)	Porosity
8	8	0.0342	2.1 × 10^−10^	4.76 × 10^−10^	0.05

**Table 2:** The seepage simulation parameters of a rock sample with a prefabricated horizontal penetrating flaw.

Kinematic Viscosity of Water, *u*(Pa·s)	Specific Yield of Rock Mass, *S*(1/m)	Permeability Coefficient, *κ*(m/s)	Damage Threshold, *c*_1_	Damage Threshold, *c*_2_
1.0 × 10^−3^	1.0 × 10^−6^	1.0 × 10^−5^	0	0.5

**Table 3:** Numerical calculation parameters of a rock sample with a single prefabricated flaw.

Rock Density, *ρ* (kg/m^3^)	Elastic Modulus, *E* (GPa)	Poisson’s Ratio, *v*	Compressive Strength, *σ_c_*	Cohesion, *c* (MPa)
2260	10.2	0.14	40.26	23.6

**Table 4:** Mechanical parameters of the sample with two parallel prefabricated flaws.

Rock Density, *ρ* (kg/m^3^)	Elastic Modulus, *E* (GPa)	Poisson’s Ratio, *v*	Tensile Strength, *σ_t_* (MPa)	Cohesion, *c* (MPa)
2650	6	0.28	23	33

**Table 5:** Mechanical parameters of a rock sample with two parallel prefabricated flaws.

Rock Density, *ρ* (kg/m^3^)	Elastic Modulus, *E* (GPa)	Poisson’s Ratio, *v*	Tensile Strength, *σ_t_* (MPa)
2600	10	0.25	1.0

## Data Availability

Not applicable.
